# A green, facile, and practical preparation of capsaicin derivatives with thiourea structure

**DOI:** 10.1038/s41598-024-61014-5

**Published:** 2024-05-08

**Authors:** Lina Chen, Zhenhua Gao, Ye Zhang, Xiandong Dai, Fanhua Meng, Yongbiao Guo

**Affiliations:** 1 State Key Laboratory of NBC Protection for Civilian, Beijing, People’s Republic of China; 2 Sichuan University of Science and Engineering, Zigong, People’s Republic of China

**Keywords:** Chemistry, Green chemistry, Organic chemistry, Chemical synthesis

## Abstract

Capsaicin derivatives with thiourea structure (CDTS) is highly noteworthy owing to its higher analgesic potency in rodent models and higher agonism in vitro. However, the direct synthesis of CDTS remains t one or more shortcomings. In this study, we present reported a green, facile, and practical synthetic method of capsaicin derivatives with thiourea structure is developed by using an automated synthetic system, leading to a series of capsaicin derivatives with various electronic properties and functionalities in good to excellent yields.

## Introduction

**Figure 1:**
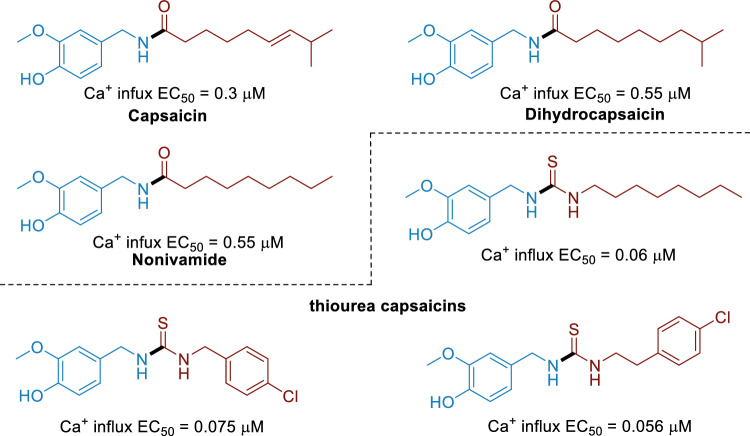
Capsaicin and its derivatives with thiourea structure.

Capsaicin is an alkaloid found in the Capsaicin family^1,2^. In general, capsaicin was used for headaches^3,4^, muscular pain^5,6^, gastroenteric protection^7,8^ and to improve circulation^9,10^. In addition, capsaicin and other capsaicinoid compounds showed strong evidence of having promising potential in the fight against many types of cancer^11–13^. Consequently, many analogues of capsaicin (Fig. 1) have been synthesized and evaluated for diverse bioactivities, among which capsaicin derivatives with thiourea structure (CDTS) is highly noteworthy owing to its higher analgesic potency in rodent models and higher agonism in vitro (Ca^2+^ influx into dorsal root ganglia neurones)^14^.

**Figure 2:**
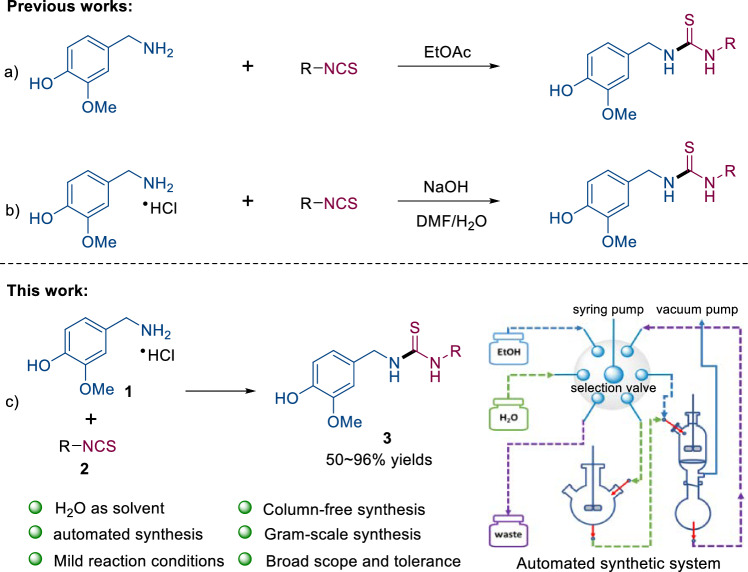
Previous studies and our work.

The pioneer work in the field was reported by Walpole et al. who employed EtOAc (Fig. 2a) or DMF (Fig. 2a) as the solvent to realize the synthesis of CDTS directly from vanilylamine or vanilylamine hydrochloride and Isothiocyanate^15,16^. However, these methodologies suffer from one or more shortcomings such as low yield, required chromatography fractionation (high consumption of organic solvents), use of toxic organic solvents (DMF, DCM and MeOH) and requirement of excess of Isothiocyanate. Therefore, further efforts are necessary in the design of novel, efficient and mild protocol wherever applicable to meet some of the green chemistry principles.

Additionally, because CDTS have strong irritancy, the synthesis and post-processing of CDTS will cause environmental pollution and make people feel uncomfortable. Recently, development of new technology that minimize pollution in chemical industry has received considerable attention due to growing environmental concerns. In this direction, with the development of automation technology, the automated synthetic systems have emerged as a useful tool to accelerate the research of organic synthesis and reduce the harm of chemicals to human body^17,18^. With these systems, several reactions were reported, including Suzuki coupling^19–21^, Buchwald-Hartwig amination^22–25^ and photordox-catalysed cross coupling^26,27^. Despite these elegant examples, an automated synthesis of CDTS has yet to be described.

Having the above points in mind, we wish to report on a green, facile, and automatic protocol for preparation of CDTS on a gram scale (Fig. 2c).

## Results and discussion

Initially, the vanilylamine hydrochloride (**1**) and 1-isothiocyanatoheptane (**2a**) were chosen as model substrates to optimize the reaction conditions, and the reaction was monitored by HPLC. The reaction of **1** and **2a** was carried out in the presence of K_2_CO_3_ in EtOH at r.t. for 24 h. The reaction proceeded smoothly to give the vanilylthiourea (**3a**) in 34% yield (Table 1, entry 1). Then, a series of solvents was evaluated for the condensation reaction (entries 2–6). The results revealed that solvent effect played a crucial role; H_2_O is the best in terms of yields. This might be attributed to the poor solubility of K_2_CO_3_ in other solvents. Encouraged by this result, subsequently, different bases, including KOH, NaOH, Na_2_CO_3_, NaHCO_3_, Na_2_SiO_3_, CeCO_3_ and NEt_3_, were examined (entries 7–13), giving good to excellent yields except weak base NaHCO_3_ (entry 10), of which Na_2_SiO_3_ offered the highest yield (entry 11). It was noteworthy that the reaction hardly occurred in absence of base (entry 14). This could be larger due to the strength of base, leading to a lower solubility (weak base) of vanilylamine hydrochloride (**1**) or a higher solubility (strong base) of vanilylthiourea (**3a**). Additional efforts at reaction optimization(base loading and reaction time)provided the similar levels of selectivity. Finally, the optimized conditions were then concluded as follows: **1** (0.2 mmol), **2a** (0.22 mmol) and Na_2_SiO_3_(0.22 mmol) in H_2_O (2 mL) at r.t. for 12 h, and 3a was obtained in 99% yield. Notably, the column chromatographic separation was not required. After simple filtration, **3a** were obtained in 84% yields.

**Table 1:** Selected optimization studies. Reaction conditions: **1a** (0.2 mmol), **2a** (0.22 or 0.4 mmol), and base (0.22 mmol), in solvent (2 mL) for 24 h.Yield was determined by HPLC analysis.


Entry	Base	Solvent	Time (h)	**1**/**2a**	Yield (%)
1	K_2_CO_3_	EtOH	24	1/2	34
2	K_2_CO_3_	CCl_4_	24	1/2	2
3	K_2_CO_3_	CH_2_Cl_2_	24	1/2	2
4	K_2_CO_3_	THF	24	1/2	4
5	K_2_CO_3_	CH_3_CN	24	1/2	14
6	K_2_CO_3_	H_2_O	24	1/2	93
7	KOH	H_2_O	24	1/2	98
8	NaOH	H_2_O	24	1/2	95
9	Na_2_CO_3_	H_2_O	24	1/2	80
10	NaHCO_3_	H_2_O	24	1/2	9
11	Na_2_SiO_3_	H_2_O	24	1/2	99
12	CeCO_3_	H_2_O	24	1/2	73
13	NEt_3_	H_2_O	24	1/2	89
14	–	H_2_O	24	1/2	1
15	Na_2_SiO_3_	H_2_O	24	1/1.1	99
16	Na_2_SiO_3_	H_2_O	12	1/1.1	99
17	Na_2_SiO_3_	H_2_O	8	1/1.1	90

**Figure 3:**
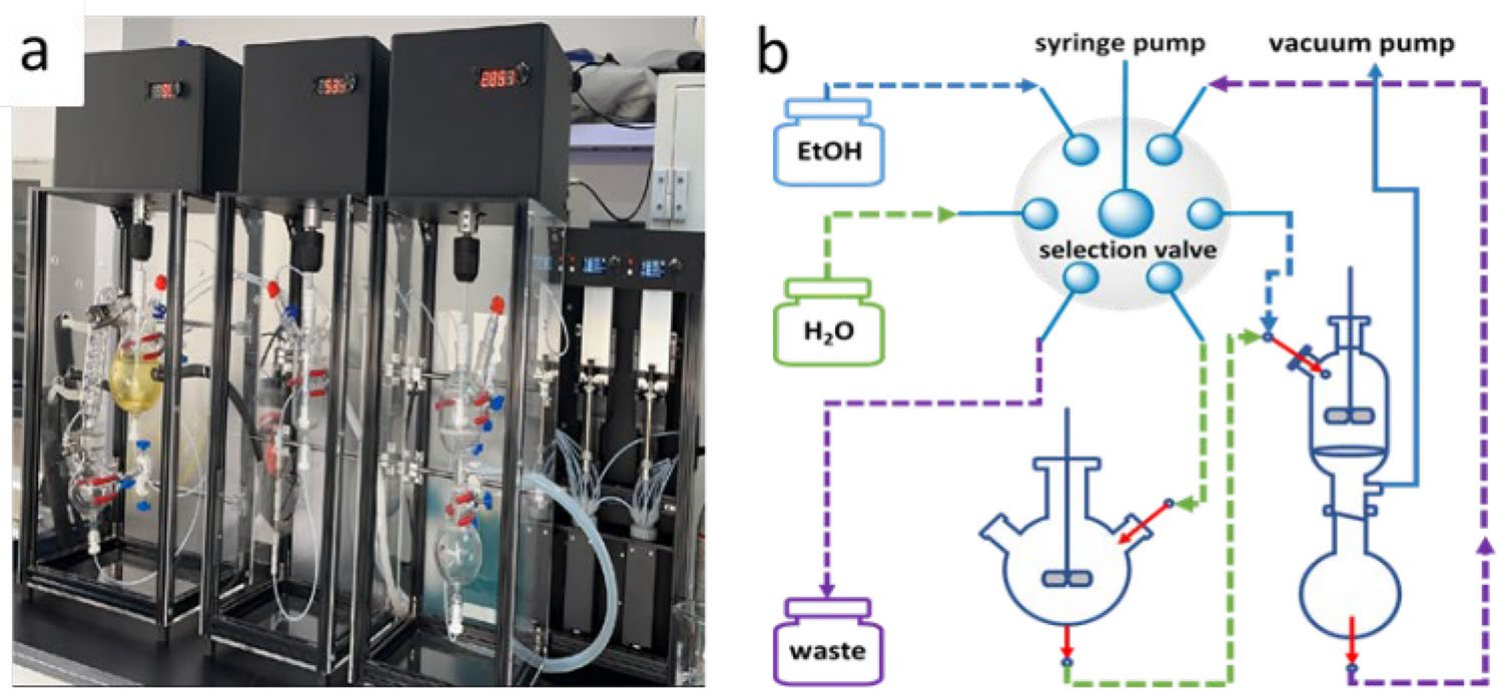
Photograph (**a**) and schematic (**b**) of the automated synthetic system.

With the potential application of this versatile synthetic transformation (green solvents, mild reaction conditions, and simple post processing) to the chemical enterprise, we explored the development of an automated synthetic system to further demonstrate the synthetic utility of this method. As depicted in Fig. 3, the automated synthetic system manly consists of seven parts: (i) central control unit; (ii) solvents; (iii) syringe pump; (iv) selection valve; (v) reaction module; (vi) filter module; (vii) vacuum pump.

**Figure 4:**
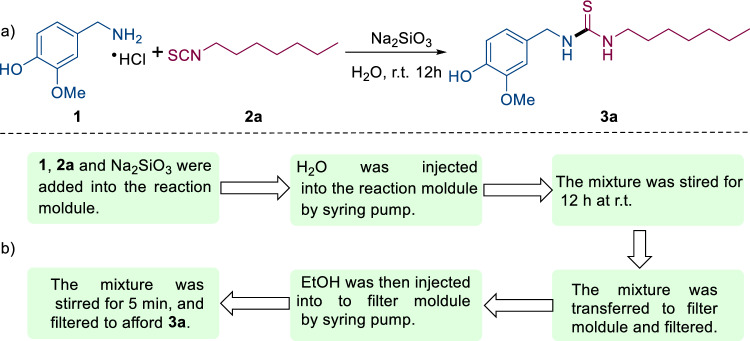
(**a**) Gram-scale synthesis of **3a**. (**b**) The representation of general six-step unit operation for **3a** synthesis.

As shown in Fig. 4a, the vanilylamine hydrochloride (**1)** and isothiocyanatoheptane (**2a**) were chosen as model substrates to validate the design concept of automated synthetic system and its potential for large scale production of thioureas with high yield. The automated synthetic system is capable of fulfilling the whole process of synthesis of **3a**, in which a general six-step sequential unit operation is included as follows (Fig. 4b): (i) **1a** (3 mmol), **2a** (3.3 mmol), and Na_2_SiO_3_ (3.3 mmol) were added into the reaction module. (ii) H_2_O (30 mL) was then injected into reaction module, which is predetermined by the program med method using the syringe pump and solvent selection value. (iii) The mixture was stired for 12 h at r.t. (iv) The mixture was transferred to filter module and filtered by vacuum pump. (v) EtOH was then injected into filter module, which is predetermined by the program med method using the syringe pump and solvent selection value. (vi) The mixture was stired for 5 min, and then filtered to give the desired thiourea **3a** in 84% yield.

**Figure 5:**
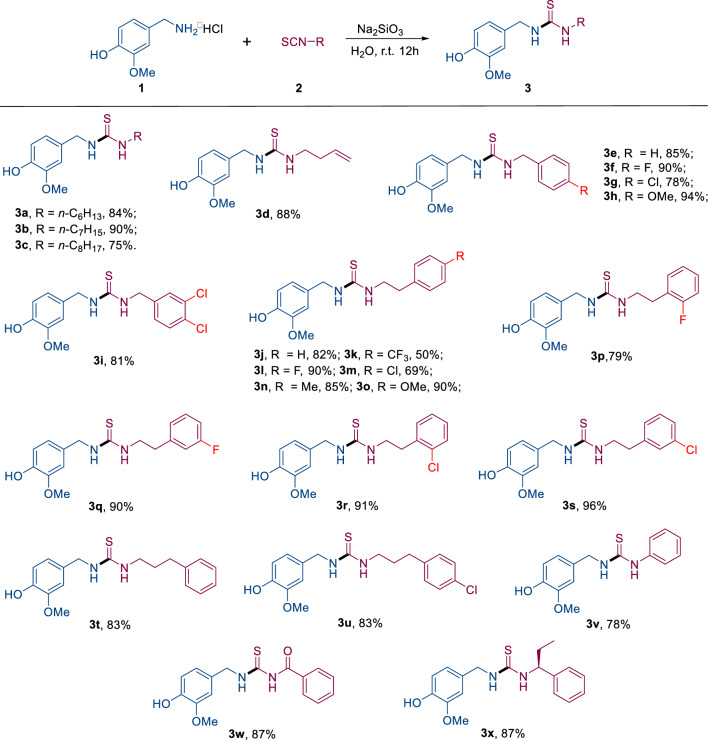
Scope of substituted isothiocyanates. Reaction conditions: **1** (3 mmol), **2** (3.3 mmol) and Na_2_SiO_3_ (3.3 mmol) in H_2_O (30 mL) for 12 h (r.t.). Filtered yields.

With the establishment of the optimized reaction conditions and the process of automated synthesis, a series of substrates was explored to determine the generality of this method, and the results are summarized in Fig. 5. In general, the corresponding thioureas **3a-3y** were obtained with good to excellent yields (69–96%), except **3 l** (50% yield). The chain length of the alkyl isothiocyanates tended to slightly influenced to the yield (**3a** vs **3b** vs **3c** vs **3d**). The substituents of benzyl isothiocyanates could contain both electron-withdrawing and electron-donating groups at the para position of benzene rings, for example, -F (**3f.**, 90% yield), -Cl (**3 g**, 78% yield), -OMe (**3 h**, 94% yield). 3,4-disubstituted benzyl isothiocyanate was viable substrate as well (**3i**, 81% yield). Under the standard conditions, product **3j** was also smoothly yielded in excellent yields (92%). In contrast to phenylethyl isothiocyanate (**2j**), substrate **2 k** bearing a strong electron withdrawing CF_3_ group at the at the para position of benzene ring led to a significantly reduction in yield (**3 g,** 82% *vs.* **3 k**, 50%), while weakly electron withdrawing group (F, and Cl), and electron donating goups (Me, and MeO) at the at the para position of benzene rings had little effect on the yield (**3 l-**3o**,** 69–90% yield). Moreover, The good tolerance of the halogen atom (F, and Cl)at different positions on the benzene rings demonstrated good compatibility of the protocol(**3 l** *vs.* **3p** *vs.* **3q**, and **3 m** *vs.* **3r** *vs.* **3 s**). After that, phenylpropyl isothiocyanates were also investigated, the reaction of substrates **2t** and **2u** readily took place under the optimized conditions, offering the corresponding products with good yields. Finally, the isothiocyanatobenzene, benzoyl isothiocyanate, and (*R*)-(1-isothiocyanatopropyl)benzene were also investigated. Products **3v**-**3 × **were smoothly yielded in 78–87% yields.

To compare the efficiency of our method with the reported methods for the synthesis of CDTS, we have tabulated the results of these methods to promote the synthesis of compounds **3a**, **3b**, **3 g**, **3j** and **3u** from. The results are summarized in Table 2. Obviously, our method showed a much higher yields and green.

**Table 2:** Comparison of our method with the reported methods for the synthesis of CDTS.

Entry	Solvent	Post-processing	Products	Yield (%)	Reference
1	H_2_O	Filtration	**3a**	78	This work
2	H_2_O		**3b**	82	
3	H_2_O		**3 g**	78	
4	H_2_O		**3j**	82	
5	H_2_O		**3u**	83	
6	DMF	Chromatography fractionation	**3a**	52	8a
7	DMF		**3b**	3	
8	DMF		**3 g**	38	8b
9	DMF		**3j**	32	
19	DMF		**3u**	44	

## Conclusions

In summary, an automated synthetic system have been have been developmented and evaluated as a equipment for the synthesis of capsaicin derivatives with thiourea structures via a condensation reaction of vanilylamine hydrochloride and isothiocyanates under room temperature and green solvent (water as solvent) conditions. The notable advantages of this method are automatic reaction and post-processing, mild reaction conditions, ready availability of starting materials, high functional groups tolerance, good to excellent yields. Thus, this procedure is a better and more practical alternative for green chemistry. Moreover, the analgesic potency in rodent models of these CDTS is being tested in our group.

## Methods

Automated synthesis of CDTS 3. (i) 1 (3 mmol), 2 (3.3 mmol), and Na2SiO3 (3.3 mmol) were added into the reaction moldule. (ii) H2O (30 mL) was then injected into reaction moldule, which is predetermined by the program med method using the syring pump and solvent selection value. (iii) The mixture was stired for 12 h at r.t. (iv) The mixture was transferred to filter moldule and filtered by vacuum pump. (v) EtOH was then injected into filter moldule, which is predetermined by the program med method using the syring pump and solvent selection value. (vi) The mixture was stired for 5 min, and then filtered to give the desired thiourea 3.

### Supplementary Information

Supplementary Information.

## Data Availability

Data is provided within the manuscript or supplementary information files.
