# Supplementary material for: A green, facile, and practical preparation of capsaicin derivatives with thiourea structure
Source: Sci Rep. 2024 May 8;14:10576. doi: 10.1038/s41598-024-61014-5 (PMC11078945; doi:10.1038/s41598-024-61014-5)
Supplement: Supplementary file 1 — Supplementary Information. [file 41598_2024_61014_MOESM1_ESM.pdf]

A green, facile, and practical preparation of capsaicin derivatives with thiourea structure

Lina Chen,[a] Zhenhua Gao,[a] Ye Zhang,[b] Xiandong Dai,[a] Fanhua Meng,[a *] Yongbiao Guo,[a*]

[a] State Key Laboratory of NBC Protection for Civilian Research, Beijing 102205, P. R. China.
[b] Sichuan University of Science & Engineering, Zigong 643000, P.R. China
E-mail: van87120@126.com;

Supporting Information

Table of Contents

1. Experimental Section and compound characterization.. 2
2. NMR spectrum of 3 10
3. References.. 33

1. Experimental Section and compound characterization

General information: Reagents and solvents were purchased from common commercial suppliers and were used without further purification. Column chromatography was generally performed on silica gel (200-300 mesh). Melting points were determined with a Büchi B-545 melting-point apparatus. 600MHz 1H NMR and 150MHz 13C NMR spectra were recorded on Varian VMS-600 spectrometers, respectively. The chemical shifts are reported in ppm (δ scale) relative to internal tetramethylsilane, and coupling constants are reported in hertz (Hz). High-resolution mass spectra (HRMS) were obtained on Agilent 6502 Q-TOF HPLC and mass spectrometry.

Automated synthesis of CDTS 3. The automated synthetic system is capable of fulfilling the whole process of synthesis of **3**, in which a general six-step sequential unit operation is included as follows (Figure 2b): (i) **1** (3 mmol), **2** (3.3 mmol), and Na_2SiO_3 (3.3 mmol) were added into the reaction moldule. (ii) H_2O (30 mL) was then injected into reaction moldule, which is predetermined by the program med method using the syring pump and solvent selection value. (iii) The mixture was stired for 12 h at r.t. (iv) The mixture was transferred to filter moldule and filtered by vacuum pump. (v) EtOH was then injected into filter moldule, which is predetermined by the program med method using the syring pump and solvent selection value. (vi) The mixture was stired for 5 min, and then filtered to give the desired thiourea **3** in 84% yield.

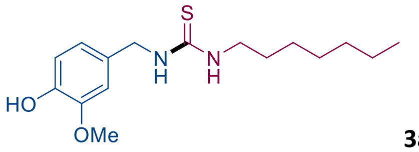

3a

1-heptyl-3-(4-hydroxy-3-methoxybenzyl)thiourea (**3a**)[1].

m.p., 91-93°C.

^{1}H NMR (600 MHz, $CDCl_3$) δ 6.87 (d, *J* = 8.0 Hz, 2H), 6.79 (dd, *J* = 8.1, 1.8 Hz, 1H), 6.04 (s, 1H), 5.82 (s, 1H), 5.67 (s, 1H), 4.54 (s, 2H), 3.87 (s, 3H), 3.34 (s, 2H), 1.56 – 1.45 (m, 2H), 1.33 – 1.15 (m, 8H), 0.86 (t, *J* = 7.1 Hz, 3H).

^{13}C NMR (151 MHz, $CDCl_3$) δ 181.49, 146.90, 145.46, 120.65 114.49 (2), 110.30, 55.97, 31.61, 28.83, 26.71, 22.50, 14.01.

HRMS (m/z) calcd for $C_{16}H_{27}N_2O_2S[M+H]^+$ 311.1793, found 311.1785.

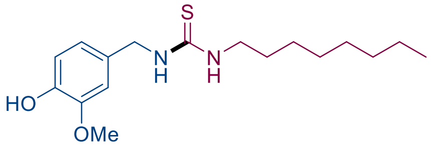

3b

1-(4-hydroxy-3-methoxybenzyl)-3-octylthiourea (**3b**)[1].

m.p., 98-100°C.

^{1}H NMR (600 MHz, $CDCl_3$) δ 6.87 (d, *J* = 8.0 Hz, 2H), 6.79 (dd, *J* = 8.1, 1.7 Hz, 1H), 6.04 (s, 1H), 5.83 (s, 1H), 5.67 (s, 1H), 4.54 (s, 2H), 3.87 (s, 3H), 3.34 (s, 2H), 1.58 – 1.45 (m, 2H), 1.32 – 1.17 (m, 10H), 0.87 (t, *J* = 7.1 Hz, 3H).

^{13}C NMR (151 MHz, $CDCl_3$) δ 181.49, 146.89, 145.45, 120.65, 114.49 (2), 110.31, 55.97, 31.70, 29.13, 29.08, 26.75, 22.57, 14.03.

HRMS (m/z) calcd for $C_{17}H_{29}N_2O_2S[M+H]^+$ 325.1950, found 325.1949.

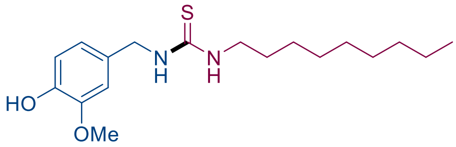

3c

1-(4-hydroxy-3-methoxybenzyl)-3-nonylthiourea (**3c**)[1].

m.p., 87-89°C.

^{1}H NMR (600 MHz, $CDCl_3$) δ 6.87 (d, *J* = 8.0 Hz, 2H), 6.80 (dd, *J* = 8.1, 1.6 Hz, 1H), 6.01 (s, 1H), 5.81 (s, 1H), 5.66 (s, 1H), 4.54 (s, 2H), 3.88 (s, 3H), 3.34 (s, 2H), 1.58 – 1.45 (m, 2H), 1.32 – 1.16 (m, 14H), 0.87 (t, *J* = 7.0 Hz, 3H).

^{13}C NMR (151 MHz, $CDCl_3$) δ 181.50, 146.90, 145.47, 120.67, 114.50 (2), 110.32, 55.98, 31.78, 29.39, 29.18, 29.17, 26.76, 22.60, 14.06.

HRMS (m/z) calcd for $C_{18}H_{31}N_2O_2S[M+H]^+$ 339.2106, found 339.2100.

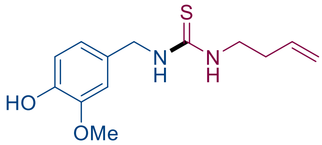

3d

1-(but-3-en-1-yl)-3-(4-hydroxy-3-methoxybenzyl)thiourea (**3d**).

m.p., 103-105°C.

^{1}H NMR (600 MHz, $CDCl_3$) δ 6.86 (t, *J* = 9.8 Hz, 2H), 6.78 (dd, *J* = 8.0, 1.8 Hz, 1H), 6.12 (s, 1H), 5.76 (s, 1H), 5.73 – 5.67 (m, 1H), 5.66 (d, *J* = 5.3 Hz, 1H), 5.05 (d, *J* = 11.6 Hz, 2H), 4.48 (s, 2H), 3.88 (s, 3H), 3.50 (s, 2H), 2.30 (q, *J* = 6.7 Hz, 2H).

^{13}C NMR (151 MHz, $CDCl_3$) δ 181.67, 146.93, 145.50, 134.58, 128.30, 120.64, 117.90, 114.51, 110.20, 55.99, 32.99.

HRMS (m/z) calcd for $C_{13}H_{19}N_2O_2S[M+H]^+$ 267.1167, found 267.1173.

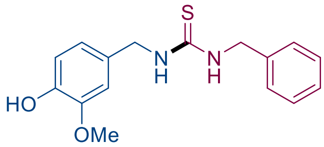

3e

1-benzyl-3-(4-hydroxy-3-methoxybenzyl)thiourea (**3e**).

m.p., 127-129°C.

^{1}H NMR (600 MHz, $CDCl_3$) δ 7.31 (dt, *J* = 13.8, 6.8 Hz, 3H), 7.23 (d, *J* = 7.0 Hz, 2H), 6.84 (d, *J* = 8.0 Hz, 1H), 6.78 (s, 1H), 6.71 (d, *J* = 7.8 Hz, 1H), 6.08 (s, 2H), 5.62 (s, 1H), 4.62 (s, 2H), 4.51 (s, 2H), 3.83 (s, 3H).

^{13}C NMR (151 MHz, $CDCl_3$) δ 181.95, 171.23, 146.90, 145.50, 128.92, 128.92, 127.98, 127.57, 120.68, 114.57, 110.21, 60.43, 55.98.

HRMS (m/z) calcd for $C_{16}H_{19}N_2O_2S[M+H]^+$ 303.1167, found 303.1170.

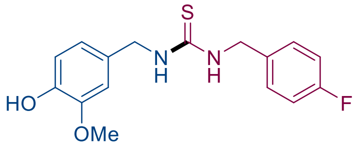

3f

1-(4-fluorobenzyl)-3-(4-hydroxy-3-methoxybenzyl)thiourea (**3f**).

m.p., 120-122°C.

^{1}H NMR (600 MHz, $CDCl_3$) δ 7.19 (dd, *J* = 8.1, 5.5 Hz, 2H), 7.02 – 6.97 (m, 2H), 6.85 (d, *J* = 8.0 Hz, 1H), 6.78 (d, *J* = 1.7 Hz, 1H), 6.73 (dd, *J* = 8.0, 1.7 Hz, 1H), 6.06 (s, 1H), 5.96 (s, 1H), 5.62 (s, 1H), 4.62 (s, 2H), 4.49 (s, 2H), 3.84 (s, 3H).

^{13}C NMR (151 MHz, $CDCl_3$) δ 181.96, 163.17, 161.54, 146.93, 145.57, 129.35, 129.29, 120.64, 115.81, 115.67, 114.58, 110.10, 55.95.

HRMS (m/z) calcd for $C_{16}H_{18}FN_2O_2S[M+H]^+$ 321.1073, found 321.1070.

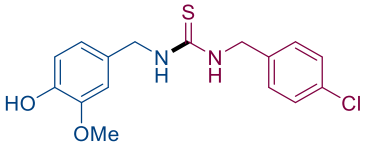

3g

1-(4-chlorobenzyl)-3-(4-hydroxy-3-methoxybenzyl)thiourea (**3g**)[2].

m.p., 136-138°C.

^{1}H NMR (600 MHz, $CDCl_3$) δ 7.27 (d, *J* = 10.2 Hz, 3H), 7.15 (d, *J* = 6.2 Hz, 2H), 6.85 (d, *J* = 7.4 Hz, 1H), 6.79 – 6.69 (m, 2H), 6.12 (s, 1H), 6.01 (s, 1H), 5.63 (s, 1H), 4.63 (s, 2H), 4.48 (s, 2H), 3.83 (s, 3H).

^{13}C NMR (151 MHz, $CDCl_3$) δ 182.04, 146.94, 145.57, 135.42, 133.71, 128.96, 128.88, 120.61, 114.58, 110.06, 55.94.

HRMS (m/z) calcd for $C_{16}H_{18}ClN_2O_2S[M+H]^+$ 337.0778, found 337.0771.

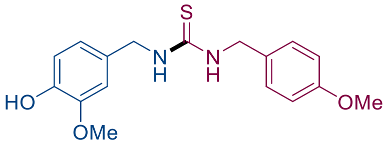

3h

1-(4-hydroxy-3-methoxybenzyl)-3-(4-methoxybenzyl)thiourea (**3h**).

m.p., 99-101°C.

^{1}H NMR (600 MHz, $CDCl_3$) δ 7.15 (d, *J* = 8.5 Hz, 2H), 6.89 – 6.81 (m, 3H), 6.77 (d, *J* = 1.8 Hz, 1H), 6.71 (dd, *J* = 8.0, 1.6 Hz, 1H), 6.02 (s, 2H), 5.62 (s, 1H), 4.52 (s, 4H), 3.83 (s, 3H), 3.79 (s, 3H).

^{13}C NMR (151 MHz, $CDCl_3$) δ 159.36, 146.87, 145.47, 128.98, 120.71, 114.53, 114.27, 110.24, 55.97, 55.32, 48.67, 48.13.

HRMS (m/z) calcd for $C_{17}H_{21}N_2O_3S[M+H]^+$ 333.1273, found 321.1070.

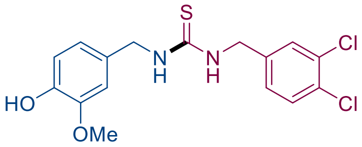

3i

1-(3,4-dichlorobenzyl)-3-(4-hydroxy-3-methoxybenzyl)thiourea (**3i**).

m.p., 169-171°C.

^{1}H NMR (600 MHz, $CDCl_3$) δ 7.36 (d, *J* = 8.2 Hz, 2H), 7.28 (s, 1H), 7.05 (d, *J* = 7.5 Hz, 2H), 6.86 (d, *J* = 8.0 Hz, 1H), 6.79 (d, *J* = 1.8 Hz, 1H), 6.75 (dd, *J* = 8.0, 1.8 Hz, 2H), 6.22 (s, 1H), 5.98 (s, 1H), 5.64 (s, 1H), 4.67 (d, *J* = 4.5 Hz, 2H), 4.48 (s, 2H), 3.85 (s, 3H).

^{13}C NMR (151 MHz, $CDCl_3$) δ 182.34, 147.06, 145.69, 137.53, 132.87, 131.83, 130.70, 129.29, 126.82, 120.55, 114.68, 109.97, 56.00, 47.52.

HRMS (m/z) calcd for $C_{16}H_{17}Cl_2N_2O_2S[M+H]^+$ 371.0188, found 371.0180.

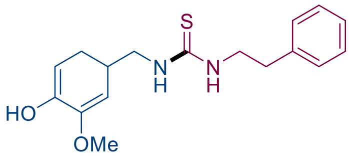

3j

1-((4-hydroxy-3-methoxycyclohexa-2,4-dien-1-yl)methyl)-3-phenethylthiourea (**3j**)[2].

m.p., 106-108°C.

^{1}H NMR (600 MHz, $CDCl_3$) δ 7.30 – 7.25 (m, 1H), 7.22 (t, J = 7.3 Hz, 1H), 7.12 (d, J = 7.3 Hz, 1H), 6.83 (d, J = 8.0 Hz, 1H), 6.76 (s, 1H), 6.67 (d, J = 7.7 Hz, 1H), 6.10 (s, 1H), 5.70 (s, 1H), 5.65 (s, 1H), 4.37 (s, 2H), 3.85 (s, 3H), 3.73 (s, 2H), 2.86 (t, J = 6.8 Hz, 2H).

^{13}C NMR (151 MHz, $CDCl_3$) δ 181.78, 146.88, 145.45 138.14, 128.74, 128.62, 126.70, 120.49, 114.52, 110.05, 55.95, 48.14, 45.69, 35.01.

HRMS (m/z) calcd for $C_{17}H_{21}N_2O_2S[M+H]^+$ 317.1324, found 317.1320.

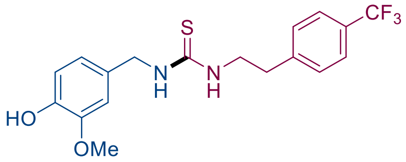

3k

1-(4-hydroxy-3-methoxybenzyl)-3-(4-(trifluoromethyl)phenethyl)thiourea (**3k**).

m.p., 147-149°C.

^{1}H NMR (600 MHz, $CDCl_3$) δ 7.52 (d, J = 8.0 Hz, 2H), 7.22 (d, J = 7.9 Hz, 2H), 6.85 (d, J = 8.0 Hz, 1H), 6.75 (s, 1H), 6.70 (d, J = 8.0 Hz, 1H), 6.14 (s, 1H), 5.63 (s, 1H), 5.59 (s, 1H), 4.37 (s, 2H), 3.84 (s, 3H), 3.80 (s, 2H), 2.93 (t, J = 6.8 Hz, 2H).

^{13}C NMR (151 MHz, $CDCl_3$) δ 182.15, 146.99, 145.60, 142.40, 128.99, 125.59, 125.57, 125.02, 123.21, 120.33, 114.61, 109.83, 55.95, 48.08, 45.48, 34.94, 29.50 (d, J = 58.1 Hz).

HRMS (m/z) calcd for $C_{18}H_{20}F_3N_2O_2S[M+H]^+$ 385.1198, found 385.1199.

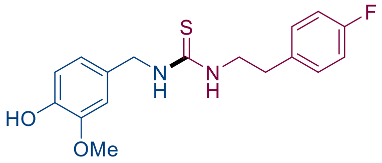

3l

1-(4-fluorophenethyl)-3-(4-hydroxy-3-methoxybenzyl)thiourea (**3l**)[2].

m.p., 150-152°C.

^{1}H NMR (600 MHz, $CDCl_3$) δ 7.07 (dd, J = 8.2, 5.5 Hz, 2H), 6.98 – 6.91 (m, 2H), 6.85 (d, J = 8.0 Hz, 1H), 6.76 (s, 1H), 6.70 (d, J = 8.0 Hz, 1H), 6.09 (s, 1H), 5.64 (s, 1H), 5.60 (s, 1H), 4.37 (s, 2H), 3.86 (s, 3H), 3.73 (d, J = 4.9 Hz, 2H), 2.83 (t, J = 6.8 Hz, 2H).

^{13}C NMR (151 MHz, $CDCl_3$) δ 181.99, 162.52, 160.90, 146.97, 145.58, 133.81, 130.11, 130.05, 120.44, 115.63, 115.49, 114.60, 109.93, 55.98, 48.14, 45.80, 34.26.

HRMS (m/z) calcd for $C_{17}H_{20}FN_2O_2S[M+H]^+$ 335.1230, found 335.1235.

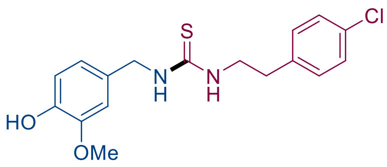

3m

1-(4-chlorophenethyl)-3-(4-hydroxy-3-methoxybenzyl)thiourea (**3m**)[2].

m.p., 144-146°C.

^{1}H NMR (600 MHz, $CDCl_3$) δ 7.25 – 7.21 (m, 2H), 7.04 (d, *J* = 8.2 Hz, 2H), 6.85 (d, *J* = 8.0 Hz, 1H), 6.75 (s, 1H), 6.70 (d, *J* = 8.0 Hz, 1H), 6.07 (s, 1H), 5.64 (s, 1H), 5.58 (s, 1H), 4.37 (s, 2H), 3.86 (s, 3H), 3.73 (s, 2H), 2.83 (t, *J* = 6.8 Hz, 2H).

^{13}C NMR (151 MHz, $CDCl_3$) δ 182.05, 146.99, 145.61, 136.64, 132.55, 129.98 (2), 128.85 (2), 120.40, 114.62, 109.87, 55.99, 48.10, 45.64, 34.43.

HRMS (m/z) calcd for $C_{17}H_{20}ClN_2O_2S[M+H]^+$ 351.0934, found 351.0930.

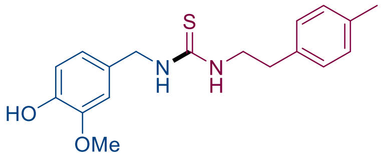

3n

1-(4-hydroxy-3-methoxybenzyl)-3-(4-methylphenethyl)thiourea (**3n**).

m.p., 116-118°C.

^{1}H NMR (600 MHz, $CDCl_3$) δ 7.08 (d, *J* = 7.8 Hz, 2H), 7.01 (d, *J* = 7.9 Hz, 2H), 6.83 (d, *J* = 8.0 Hz, 1H), 6.77 (s, 1H), 6.67 (d, *J* = 7.7 Hz, 1H), 6.01 (s, 1H), 5.67 (s, 1H), 5.63 (s, 1H), 4.38 (s, 2H), 3.86 (s, 3H), 3.69 (s, 2H), 2.81 (t, *J* = 6.8 Hz, 2H), 2.31 (s, 3H).

^{13}C NMR (151 MHz, $CDCl_3$) δ 181.77, 146.88, 145.47, 136.33, 134.95, 129.44 (2), 128.48 (2), 120.53, 114.49 (2), 110.05, 55.95, 48.46, 45.76, 34.55, 21.00.

HRMS (m/z) calcd for $C_{18}H_{23}N_2O_2S[M+H]^+$ 331.1480, found 331.1485.

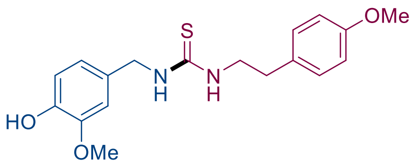

3o

1-(4-hydroxy-3-methoxybenzyl)-3-(4-methoxyphenethyl)thiourea (**30**).

m.p., 121-123°C.

^{1}H NMR (600 MHz, $CDCl_3$) δ 7.03 (d, *J* = 8.5 Hz, 2H), 6.84 (d, *J* = 8.0 Hz, 1H), 6.82 – 6.78 (m, 2H), 6.77 (s, 1H), 6.68 (d, *J* = 7.8 Hz, 1H), 6.02 (s, 1H), 5.67 (s, 1H), 5.63 (s, 1H), 4.38 (s, 2H), 3.86 (s, 3H), 3.78 (s, 3H), 3.69 (s, 2H), 2.79 (t, *J* = 6.8 Hz, 2H).

^{13}C NMR (151 MHz, $CDCl_3$) δ 181.79, 158.40, 146.89, 145.47, 130.00, 129.57 (2), 120.49, 114.53 (2), 114.16 (2), 110.05, 99.96, 55.96, 55.24, 48.25, 45.63, 34.10.

HRMS (m/z) calcd for $C_{18}H_{23}N_2O_3S[M+H]^+$ 347.1429, found 347.1426.

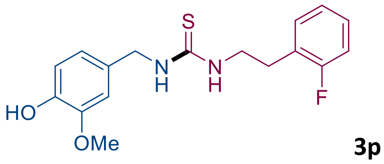

3p

1-(2-fluorophenethyl)-3-(4-hydroxy-3-methoxybenzyl)thiourea (**3p**).

m.p., 1131-133°C.

^{1}H NMR (600 MHz, $CDCl_3$) δ 7.21 (tdd, *J* = 7.3, 5.4, 1.8 Hz, 1H), 7.13 (td, *J* = 7.5, 1.4 Hz, 1H), 7.05 (td, *J* = 7.5, 1.1 Hz, 1H), 7.02 – 6.97 (m, 1H), 6.86 – 6.82 (m, 1H), 6.79 (s, 1H), 6.72 (d, *J* = 8.0 Hz, 1H), 6.08 (s, 1H), 5.75 (s, 1H), 5.63 (s, 1H), 4.42 (s, 2H), 3.86 (s, 3H), 3.73 (s 2H), 2.92 (t, *J* = 6.9 Hz, 2H).

^{13}C NMR (151 MHz, $CDCl_3$) δ 181.92, 161.95, 160.33, 146.89, 145.49, 131.02 (d, *J* = 4.7 Hz), 128.57 (d, *J* = 8.2 Hz), 124.38 (d, *J* = 3.5 Hz), 120.56, 115.40 (d, *J* = 22.0 Hz), 114.55, 110.06, 55.96, 48.22, 44.59, 29.49 (d, *J* = 57.8 Hz), 28.60.

HRMS (m/z) calcd for $C_{17}H_{20}FN_2O_2S[M+H]^+$ 335.1230, found 335.1232.

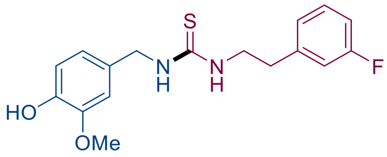

3q

1-(3- fluorophenethyl)-3-(4-hydroxy-3-methoxybenzyl)thiourea (**3q**).

m.p., 100-102°C.

^{1}H NMR (600 MHz, $CDCl_3$) δ 7.22 (td, *J* = 7.9, 6.1 Hz, 1H), 6.94 – 6.87 (m, 2H), 6.84 (dd, *J* = 7.1, 5.0 Hz, 2H), 6.77 (s, 1H), 6.69 (d, *J* = 7.7 Hz, 1H), 6.13 (s, 1H), 5.64 (s, 2H), 4.37 (s, 2H), 3.86 (s, 3H), 3.74 (d, *J* = 10.1 Hz, 2H), 2.86 (t, *J* = 6.8 Hz, 2H).

^{13}C NMR (151 MHz, $CDCl_3$) δ 182.04, 163.77, 162.14, 146.99 145.56, 140.80, 130.25 (d, *J* = 8.3 Hz), 124.31 (d, *J* = 2.8 Hz), 120.45, 115.54 (d, *J* = 21.0 Hz), 114.59, 113.67 (d, *J* = 21.0 Hz), 109.95, 55.98, 48.16, 45.49, 34.79, 29.52 (d, *J* = 57.2 Hz).

HRMS (m/z) calcd for $C_{17}H_{20}FN_2O_2S[M+H]^+$ 335.1230, found 335.1229.

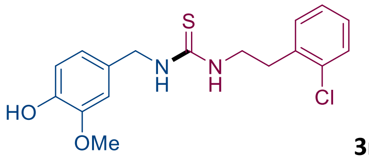

3r

1-(2-chlorophenethyl)-3-(4-hydroxy-3-methoxybenzyl)thiourea (**3r**).

m.p., 141-143°C.

^{1}H NMR (600 MHz, $CDCl_3$) δ 7.32 (tt, *J* = 4.9, 2.4 Hz, 1H), 7.17 (d, *J* = 3.0 Hz, 3H), 6.85 (d, *J* = 8.0 Hz, 1H), 6.80 (s, 1H), 6.74 (d, *J* = 7.9 Hz, 1H), 6.04 (s, 1H), 5.77 (s, 1H), 5.62 (s, 1H), 4.44 (s, 2H), 3.87 (s, 3H), 3.72 (s, 2H), 3.02 (t, *J* = 7.0 Hz, 2H).

^{13}C NMR (151 MHz, $CDCl_3$) δ 181.87, 146.88, 145.51, 133.91, 130.94 (2), 129.65 (2), 128.31, 127.17, 120.70, 114.56, 110.17, 55.98, 48.03, 43.79, 32.85.

HRMS (m/z) calcd for $C_{17}H_{20}ClN_2O_2S[M+H]^+$ 351.0934, found 351.0937.

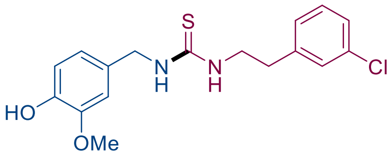

3s

1-(3-chlorophenethyl)-3-(4-hydroxy-3-methoxybenzyl)thiourea (**3s**).

m.p., 121-122°C.

^{1}H NMR (600 MHz, $CDCl_3$) δ 7.21 – 7.17 (m, 2H), 7.15 (s, 1H), 7.00 (d, *J* = 3.8 Hz, 1H), 6.84 (d, *J* = 8.0 Hz, 1H), 6.77 (s, 1H), 6.70 (d, *J* = 7.7 Hz, 1H), 6.11 (s, 1H), 5.63 (s, 2H), 4.38 (s, 2H), 3.86 (s, 3H), 3.74 (d, *J* = 6.0 Hz, 2H), 2.85 (t, *J* = 6.9 Hz, 2H).

^{13}C NMR (151 MHz, $CDCl_3$) δ 182.05, 146.98, 145.56, 140.29, 134.49, 130.02 (2), 128.78, 126.92, 126.91, 120.47, 114.61, 109.96, 56.00, 48.06, 45.44, 34.72.

HRMS (m/z) calcd for $C_{17}H_{20}ClN_2O_2S[M+H]^+$ 351.0934, found 351.0925.

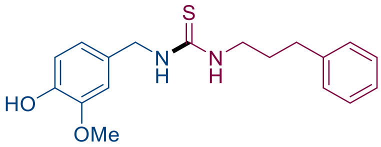

3t

1-(4-hydroxy-3-methoxybenzyl)-3-(3-phenylpropyl)thiourea (**3t**)[2].

m.p., 99-101°C.

^{1}H NMR (600 MHz, $CDCl_3$) δ 7.30 – 7.24 (m, 2H), 7.19 (t, *J* = 7.4 Hz, 1H), 7.15 – 7.11 (m, 2H), 6.88 (d, *J* = 8.0 Hz, 1H), 6.84 (s, 1H), 6.77 (dd, *J* = 8.0, 1.9 Hz, 1H), 5.91 (s, 1H), 5.70 (s, 1H), 5.63 (s, 1H), 4.44 (s, 2H), 3.87 (s, 3H), 3.42 (s, 2H), 2.62 (t, *J* = 7.5 Hz, 2H), 1.96 – 1.84 (m, 2H).

^{13}C NMR (151 MHz, $CDCl_3$) δ 181.63, 146.95, 145.56, 140.86, 128.60 (2), 128.34 (2), 126.23, 120.70, 114.56, 110.27, 56.02, 48.48, 43.81, 33.04, 30.30.

HRMS (m/z) calcd for $C_{18}H_{23}N_2O_2S[M+H]^+$ 331.1480, found 331.1478.

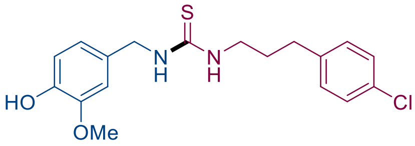

3u

1-(3-(4-chlorophenyl)propyl)-3-(4-hydroxy-3-methoxybenzyl)thiourea (**3u**) [2].

m.p., 113-115°C.

^{1}H NMR (600 MHz, $CDCl_3$) δ 7.26 (s, 1H), 7.25 – 7.21 (m, 1H), 7.05 (d, *J* = 8.4 Hz, 2H), 6.89 (d, *J* = 8.0 Hz, 1H), 6.84 (s, 1H), 6.79 (dd, *J* = 8.0, 1.9 Hz, 1H), 5.96 (s, 1H), 5.68 (s, 1H), 5.64 (s, 1H), 4.45 (s, 2H), 3.88 (s, 3H), 3.43 (s, 2H), 2.58 (t, *J* = 7.5 Hz, 2H), 1.91 – 1.82 (m, 3H).

^{13}C NMR (151 MHz, $CDCl_3$) δ 181.74, 146.97, 145.58, 139.25, 139.25, 131.93, 129.63 (2), 128.64 (2), 120.63, 114.58, 110.15, 56.01, 48.26, 43.80, 32.29, 30.26.

HRMS (m/z) calcd for $C_{18}H_{22}ClN_2O_2S[M+H]^+$ 365.1091, found 365.1099.

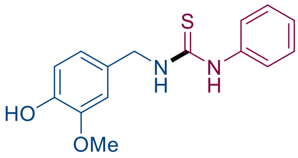

3v

1-(4-hydroxy-3-methoxybenzyl)-3-phenylthiourea (**3a**).

m.p., 1137-139°C.

^{1}H NMR (600 MHz, $CDCl_3$) δ 7.82 (s, 1H), 7.39 (t, *J* = 7.7 Hz, 2H), 7.28 (d, *J* = 7.5 Hz, 1H), 7.18 (d, *J* = 7.7 Hz, 2H), 6.92 – 6.80 (m, 2H), 6.75 (d, *J* = 7.3 Hz, 1H), 6.19 (s, 1H), 5.60 (s, 1H), 4.78 (d, *J* = 5.1 Hz, 2H), 3.87 (s, 3H).

^{13}C NMR (151 MHz, $CDCl_3$) δ 180.60, 146.66, 145.30, 135.84, 130.27, 129.03, 127.46, 125.24 (2), 120.76, 114.41, 110.57, 55.95, 49.51.

HRMS (m/z) calcd for $C_{15}H_{17}N_2O_2S[M+H]^+$ 289.1011, found 289.1015.

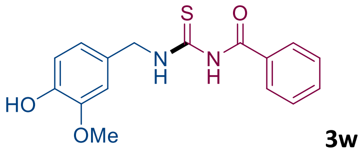

3w

N-((4-hydroxy-3-methoxybenzyl)carbamothioyl)benzamide (**3w**).

m.p., 153-155°C.

^{1}H NMR (600 MHz, $CDCl_3$) δ 10.92 (s, 1H), 8.99 (d, s, 1H), 7.83 (dt, *J* = 8.5, 1.6 Hz, 2H), 7.65 – 7.60 (m, 1H), 7.54 – 7.49 (m, 2H), 6.93 (d, *J* = 1.0 Hz, 1H), 6.90 (t, *J* = 1.2 Hz, 2H), 5.65 (s, 1H), 4.83 (d, *J* = 5.3 Hz, 2H), 3.91 (s, 3H).

^{13}C NMR (151 MHz, $CDCl_3$) δ 179.63, 166.72, 146.71, 145.48, 133.60, 131.70, 129.16 (2), 127.99, 127.38 (2), 121.26, 114.57, 110.79, 55.98, 49.87.

HRMS (m/z) calcd for $C_{16}H_{17}N_2O_3S[M+H]^+$ 317.0960, found 317.0956.

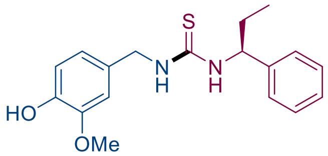

3x

(S)-1-(4-hydroxy-3-methoxybenzyl)-3-(1-phenylpropyl)thiourea (**3x**).

m.p., 119-121°C.

^{1}H NMR (600 MHz, $CDCl_3$) δ 7.33 – 7.29 (m, 2H), 7.28 (dt, *J* = 5.1, 2.0 Hz, 1H), 7.20 (d, *J* = 7.2 Hz, 2H), 6.77 (d, *J* = 8.0 Hz, 1H), 6.60 (s, 1H), 6.47 (s, 1H), 6.29 (s, 1H), 5.65 (s, 1H), 5.57 (s, 1H), 4.63 – 4.37 (m, 3H), 3.78 (s, 3H), 1.90 – 1.68 (m, 2H), 0.87 (t, *J* = 7.4 Hz, 3H).

^{13}C NMR (151 MHz, $CDCl_3$) δ 181.11, 146.68, 145.22, 140.51, 129.07, 128.03, 126.31 (2), 120.32 (2), 114.36, 110.06, 59.95, 55.90, 48.87, 30.42, 10.36.

HRMS (m/z) calcd for $C_{18}H_{23}N_2O_2S[M+H]^+$ 331.1480, found 331.1482.

2. NMR spectrum of **3**

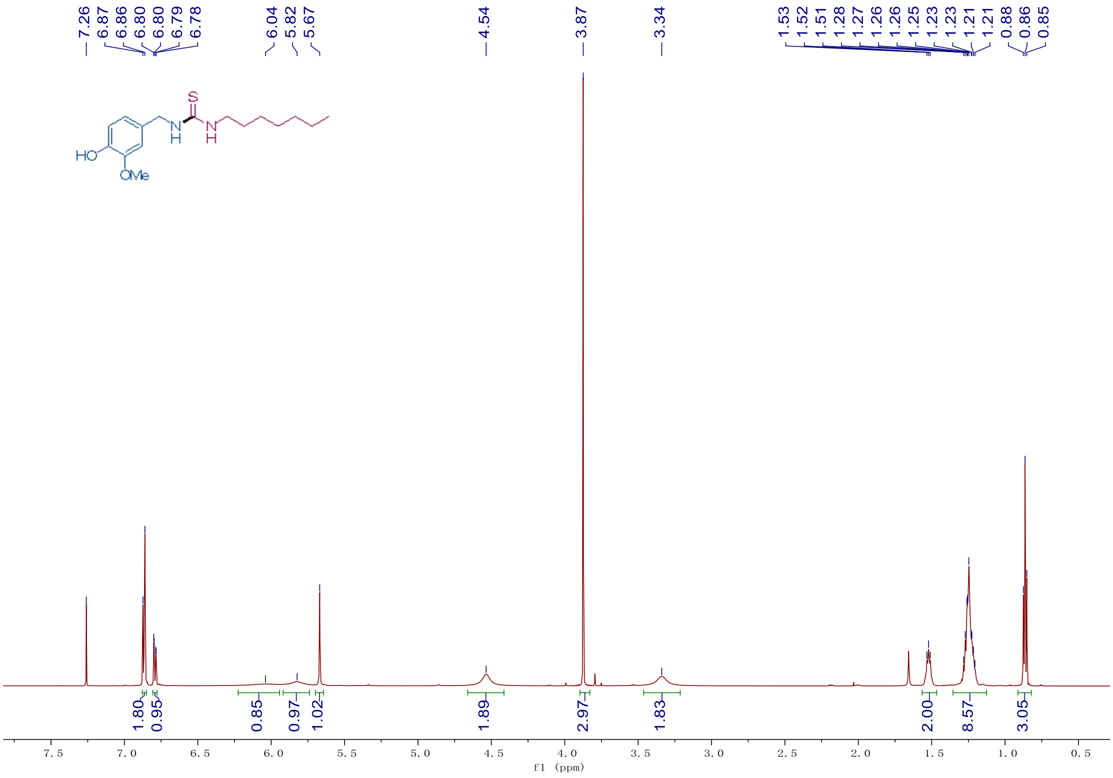

^{1}H NMR of compound **3a**

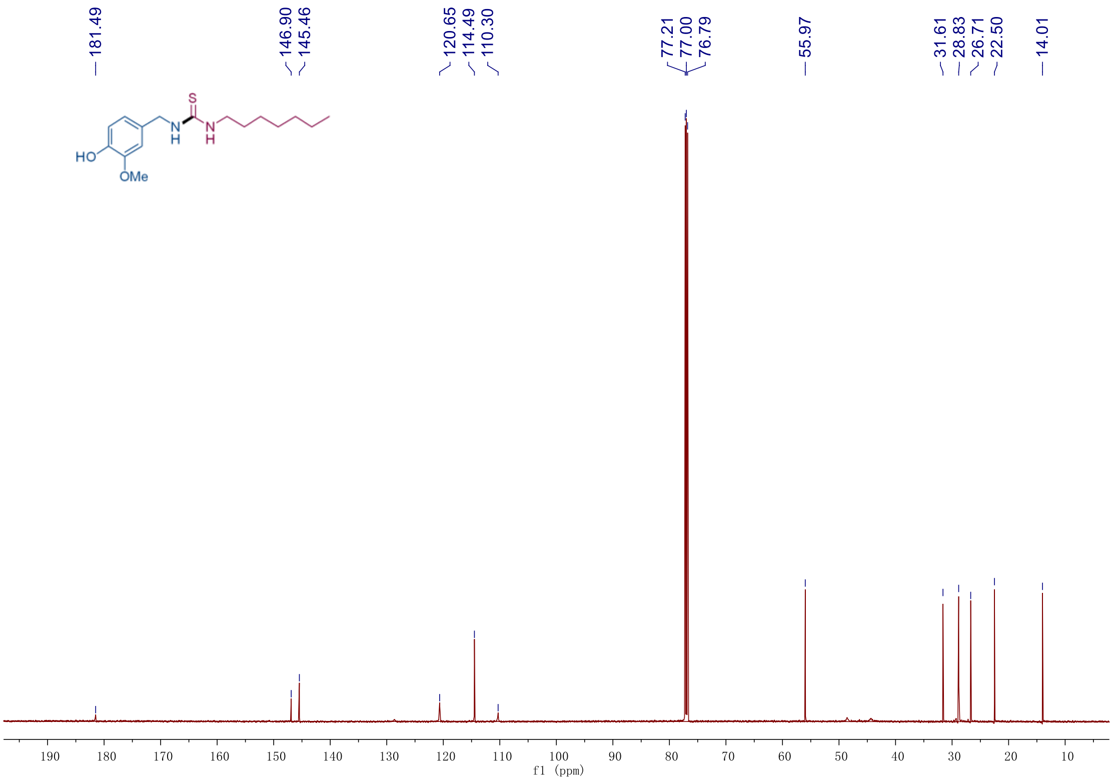

^{13}C NMR of compound **3a**

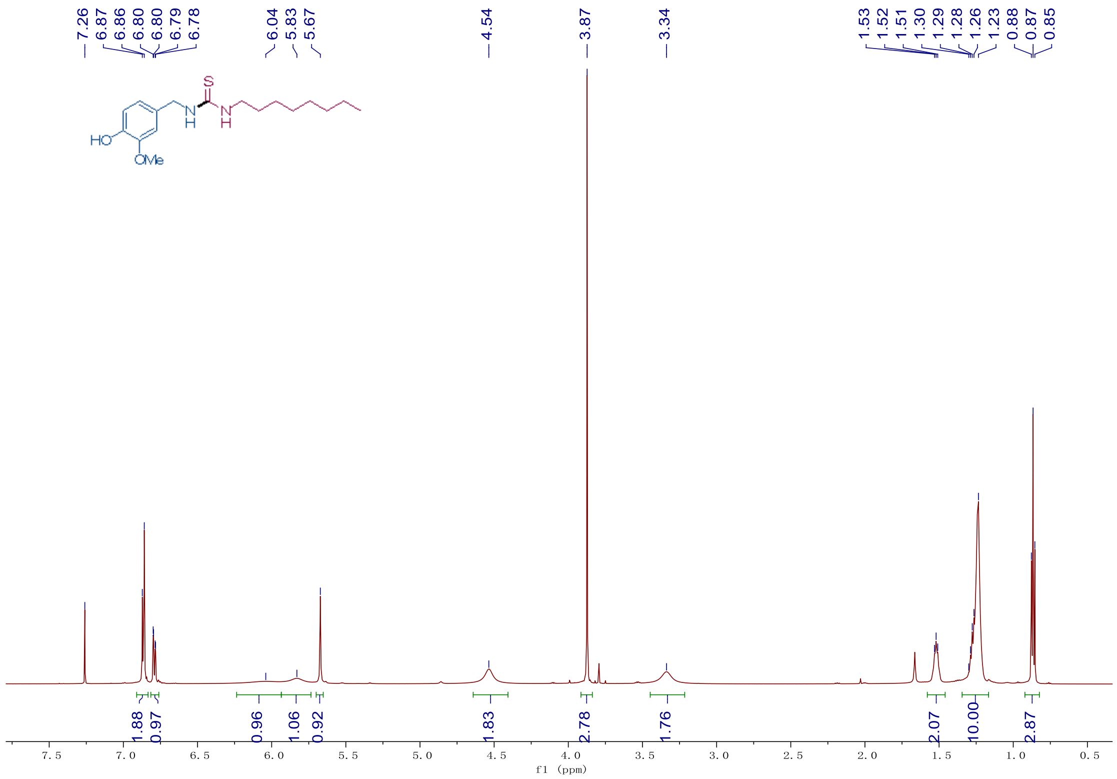

^{1}H NMR of compound **3b**

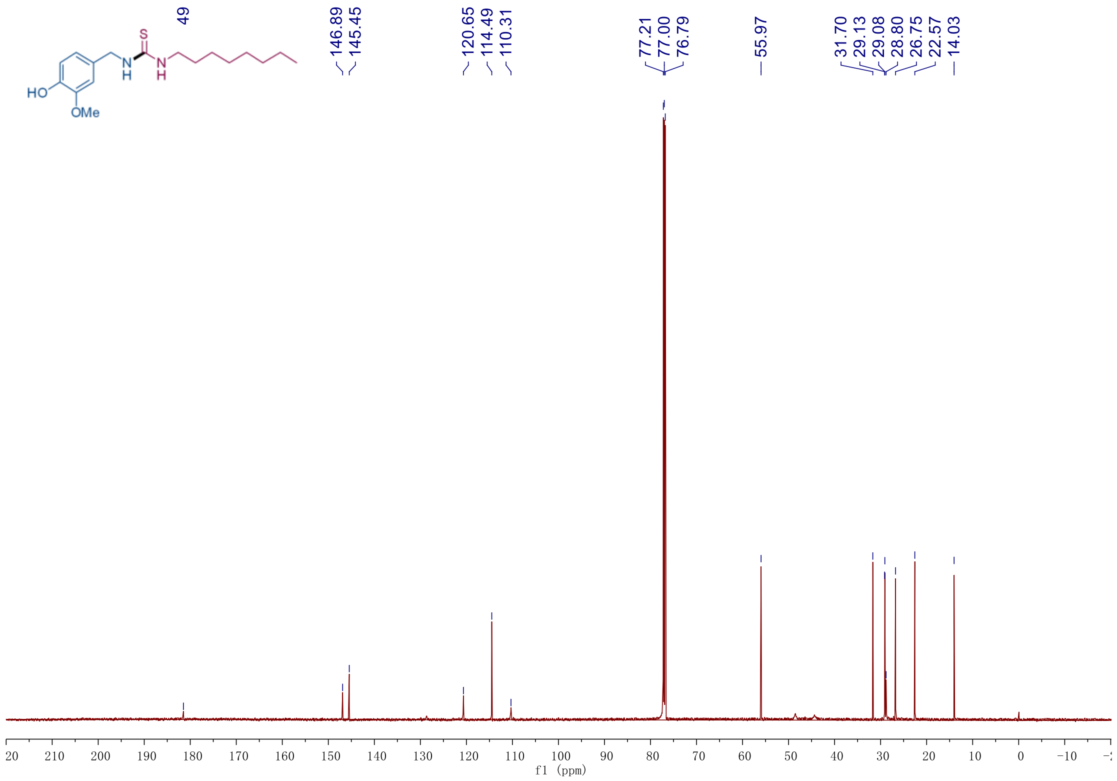

^{13}C NMR of compound **3b**

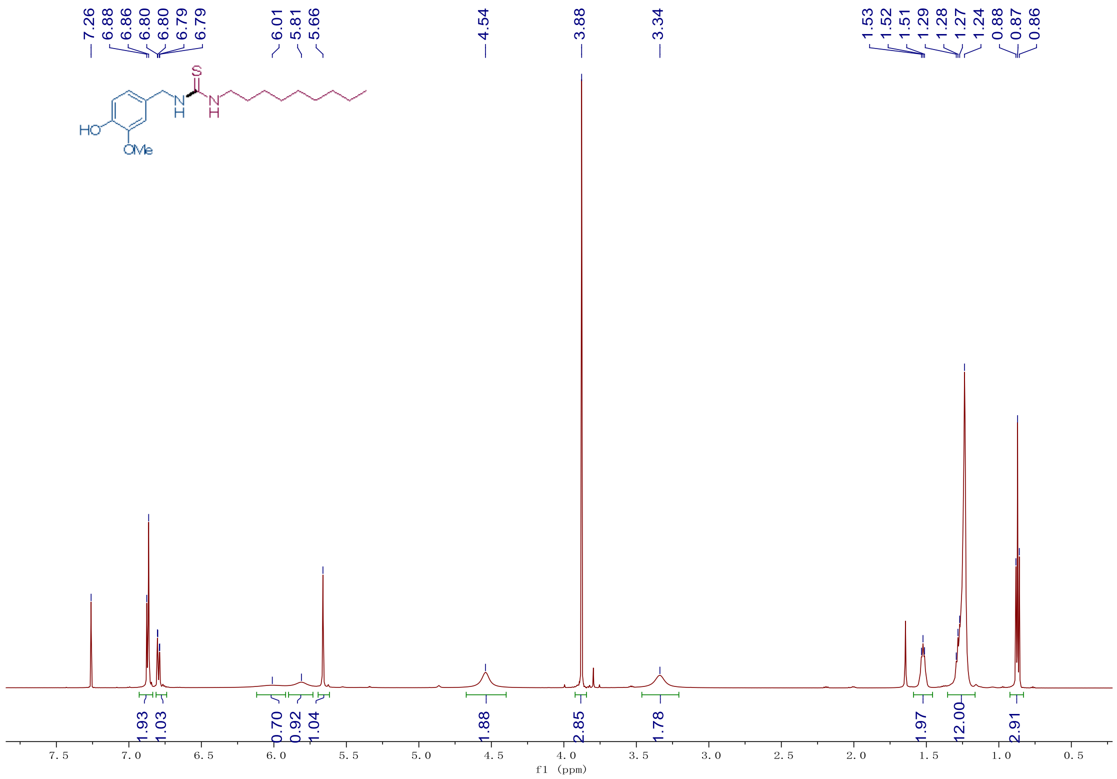

^{1}H NMR of compound **3c**

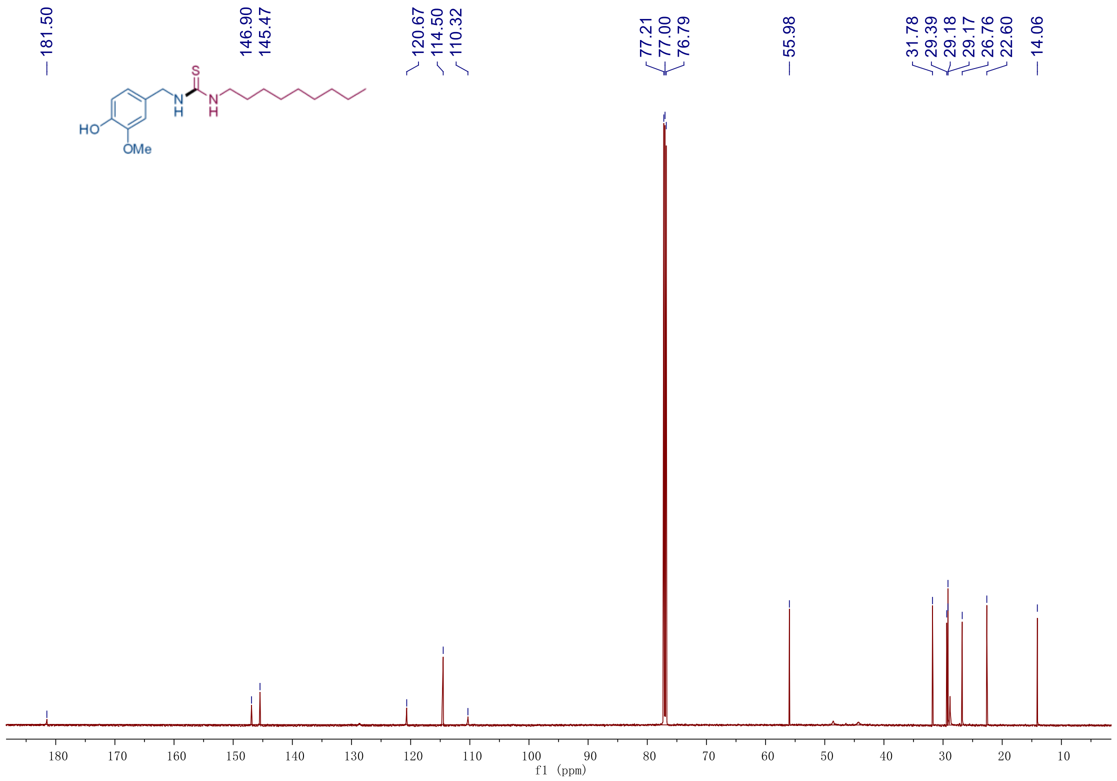

^{13}C NMR of compound **3c**

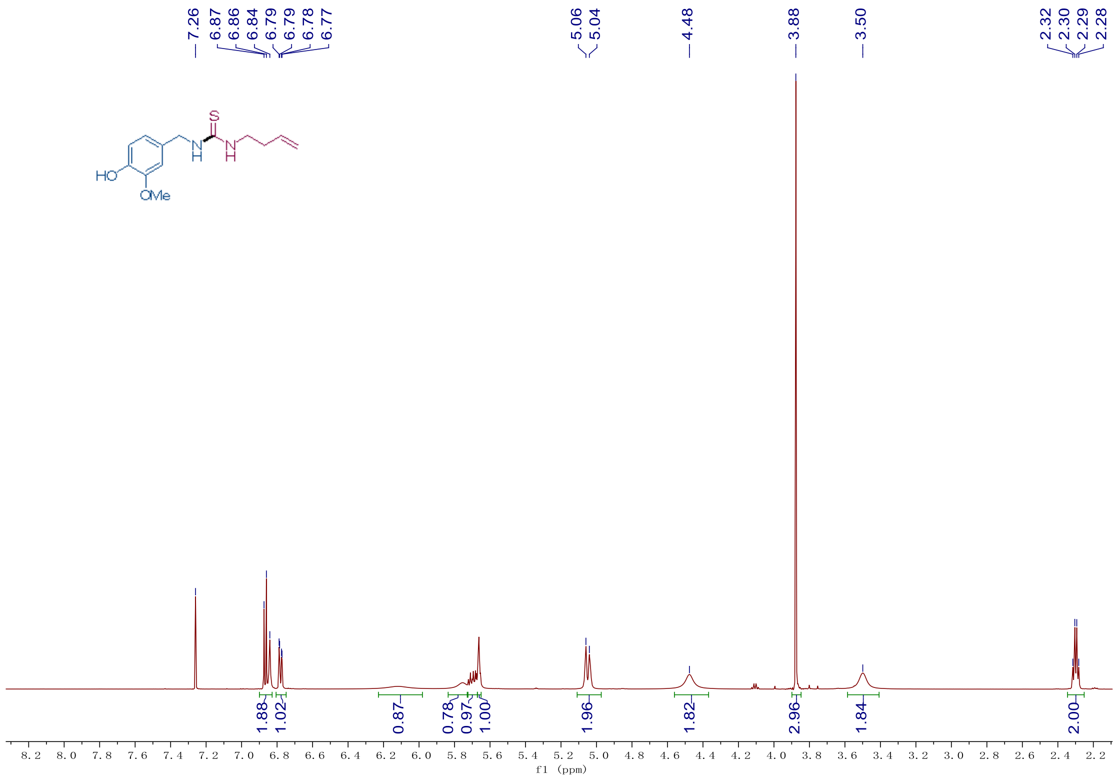

^{1}H NMR of compound **3d**

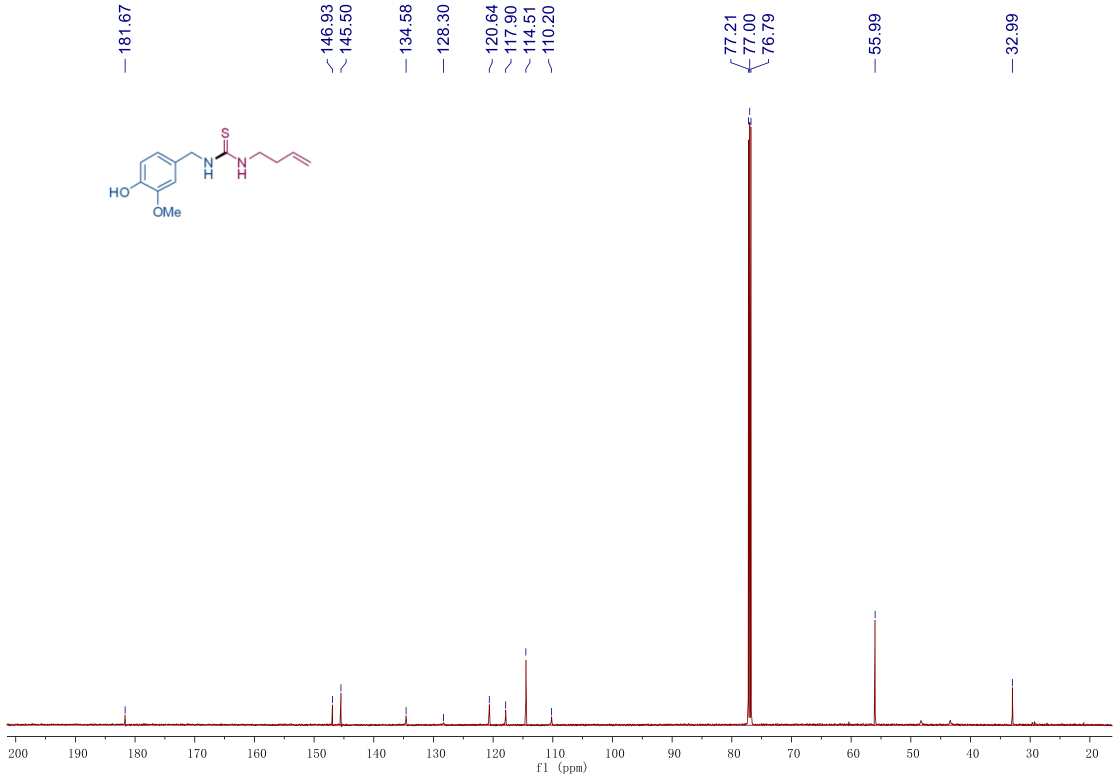

^{13}C NMR of compound **3d**

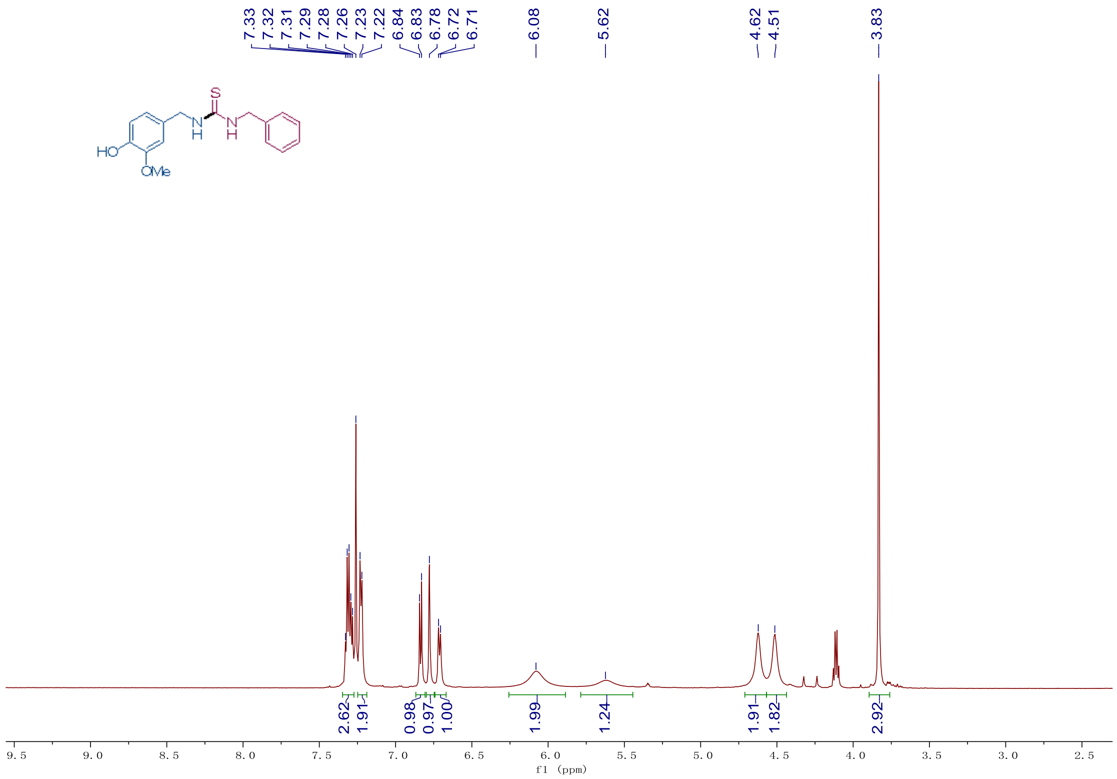

^{1}H NMR of compound **3e**

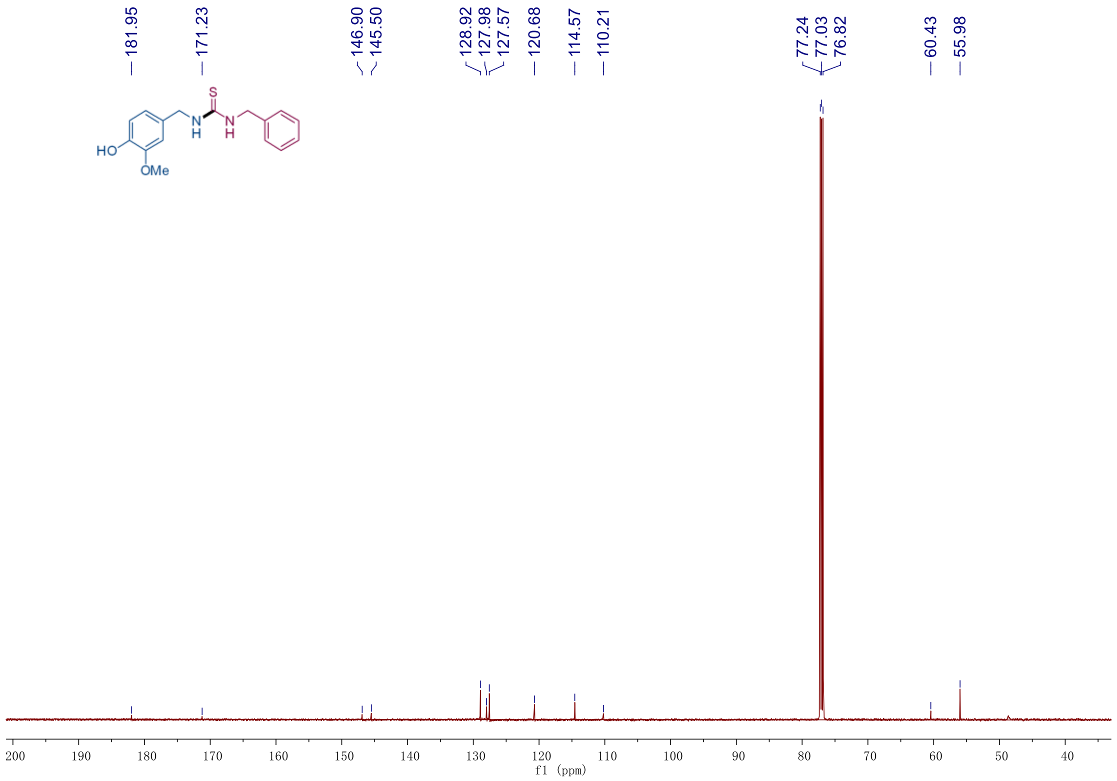

^{13}C NMR of compound **3e**

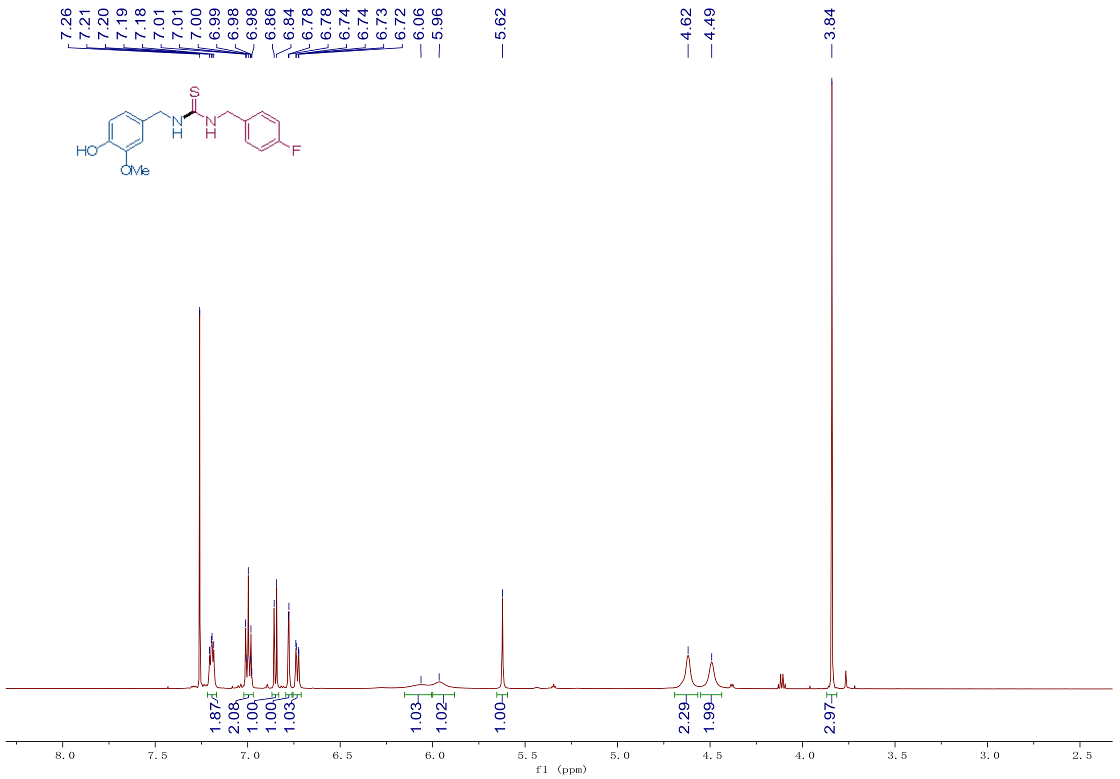

^{1}H NMR of compound **3f**

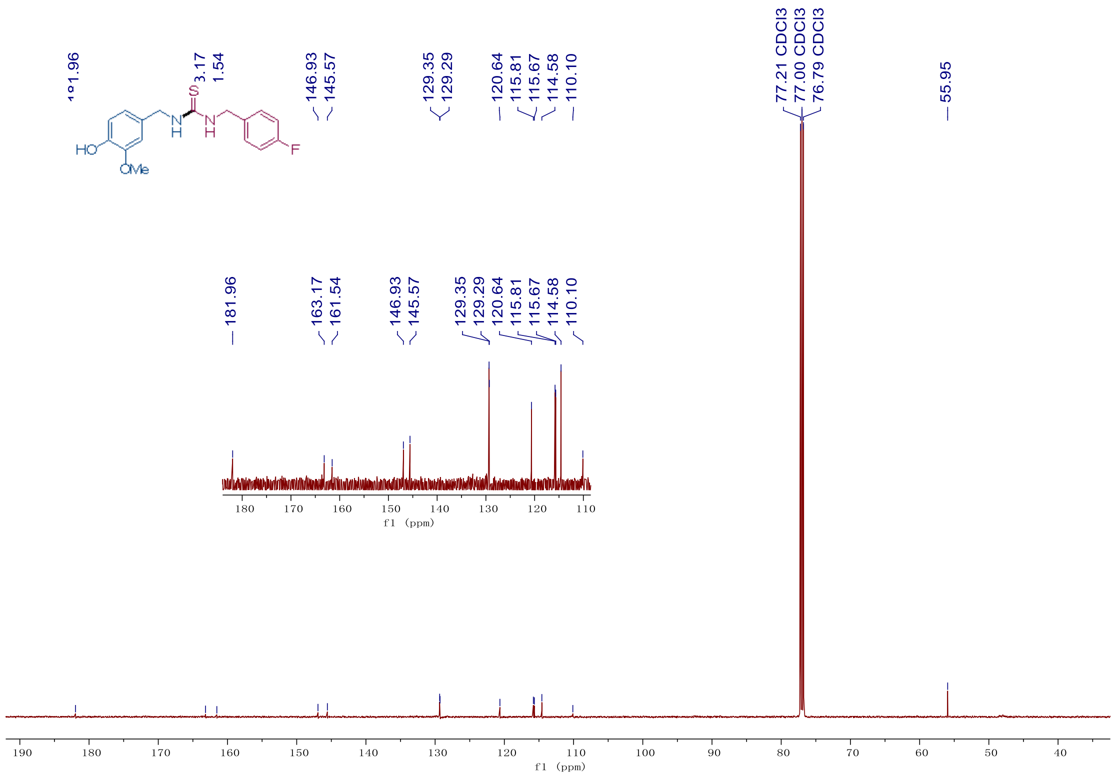

^{13}C NMR of compound **3f**

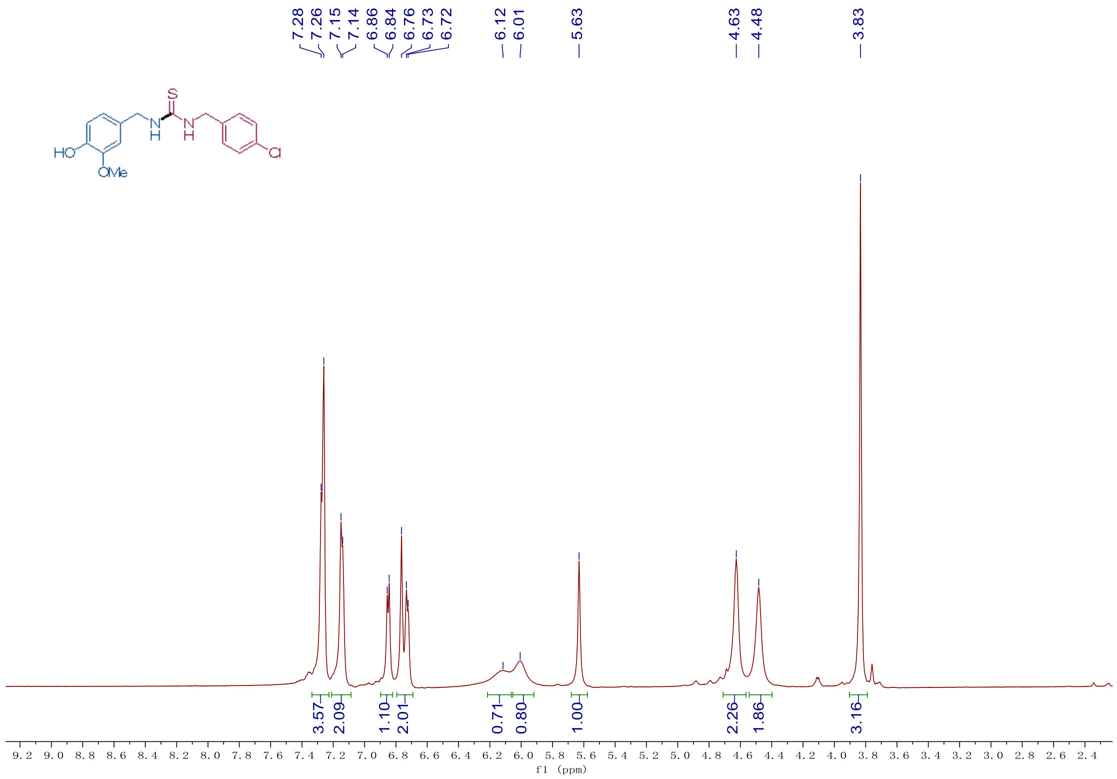

^{1}H NMR of compound **3g**

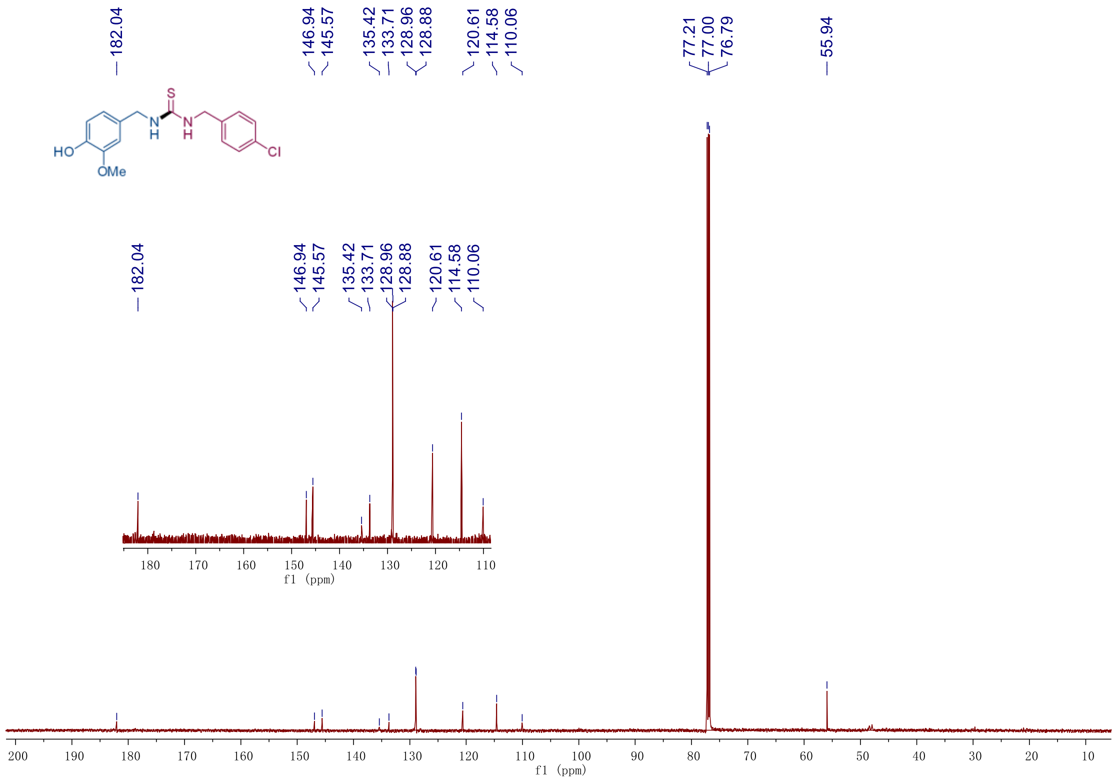

^{13}C NMR of compound **3g**

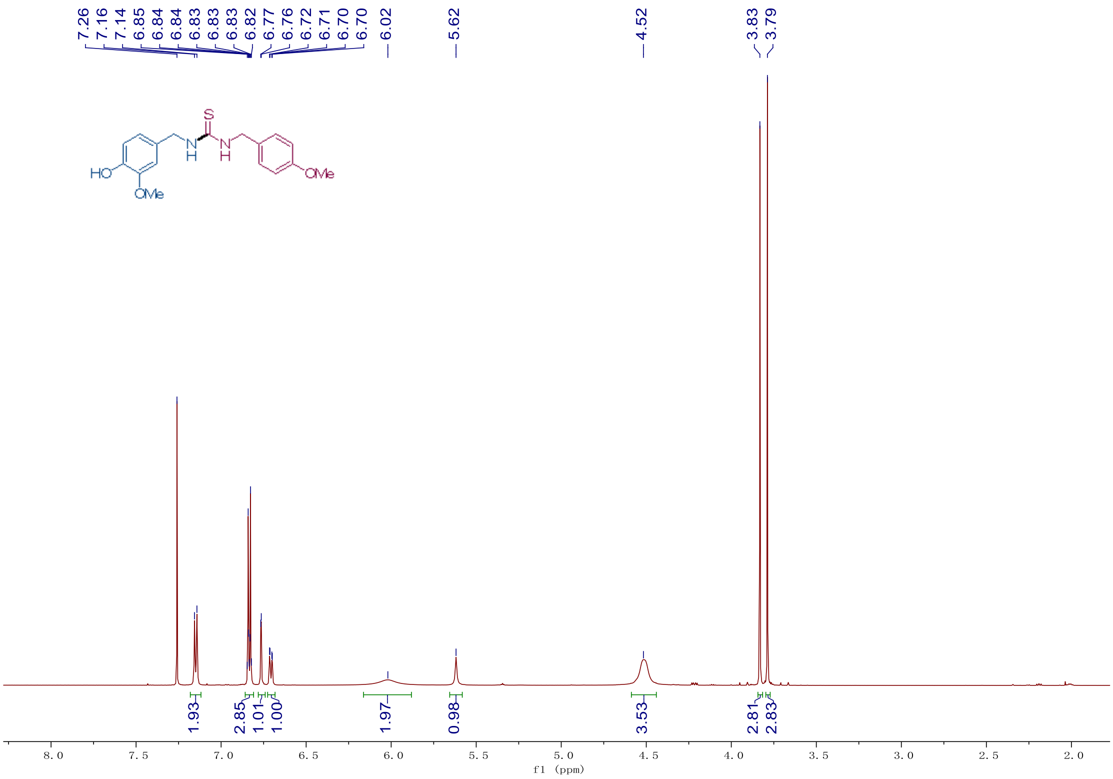

^{1}H NMR of compound **3h**

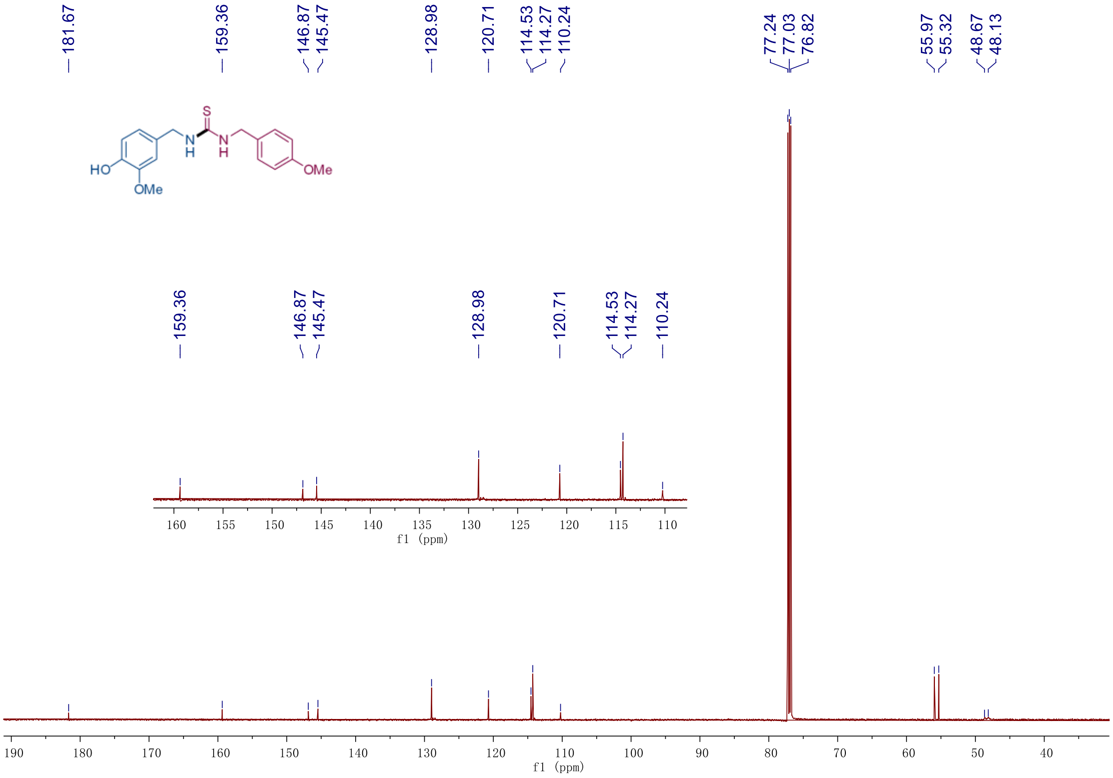

^{13}C NMR of compound **3h**

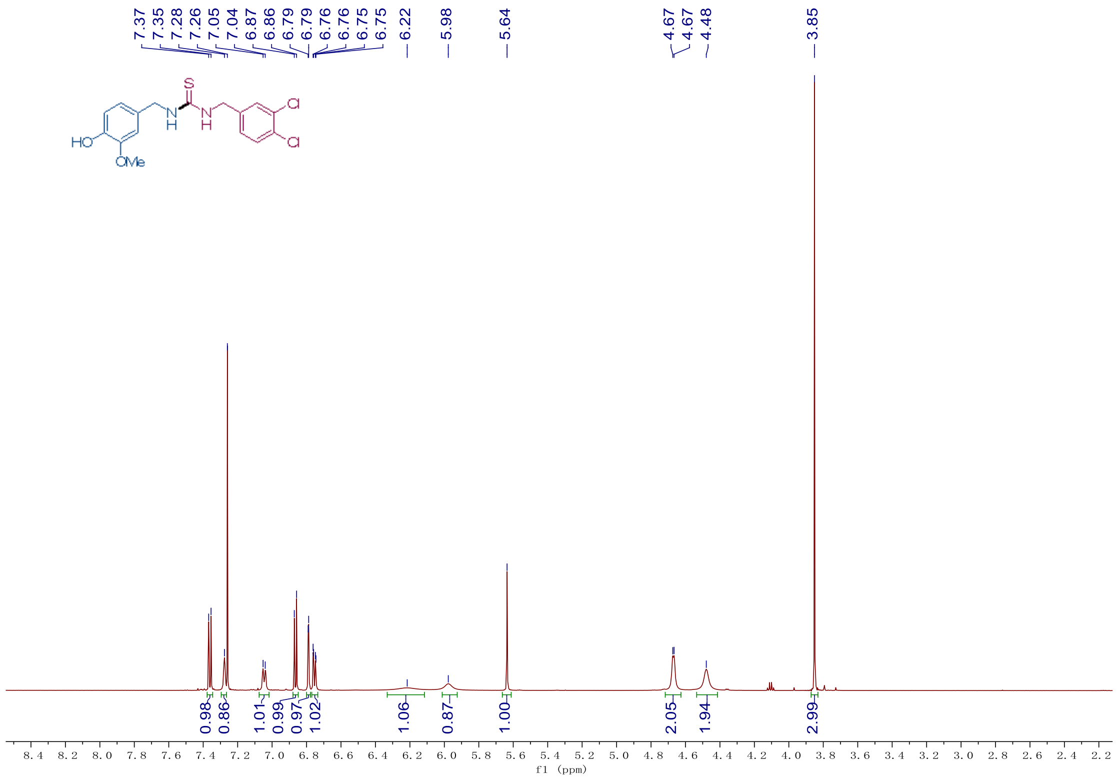

^{1}H NMR of compound **3i**

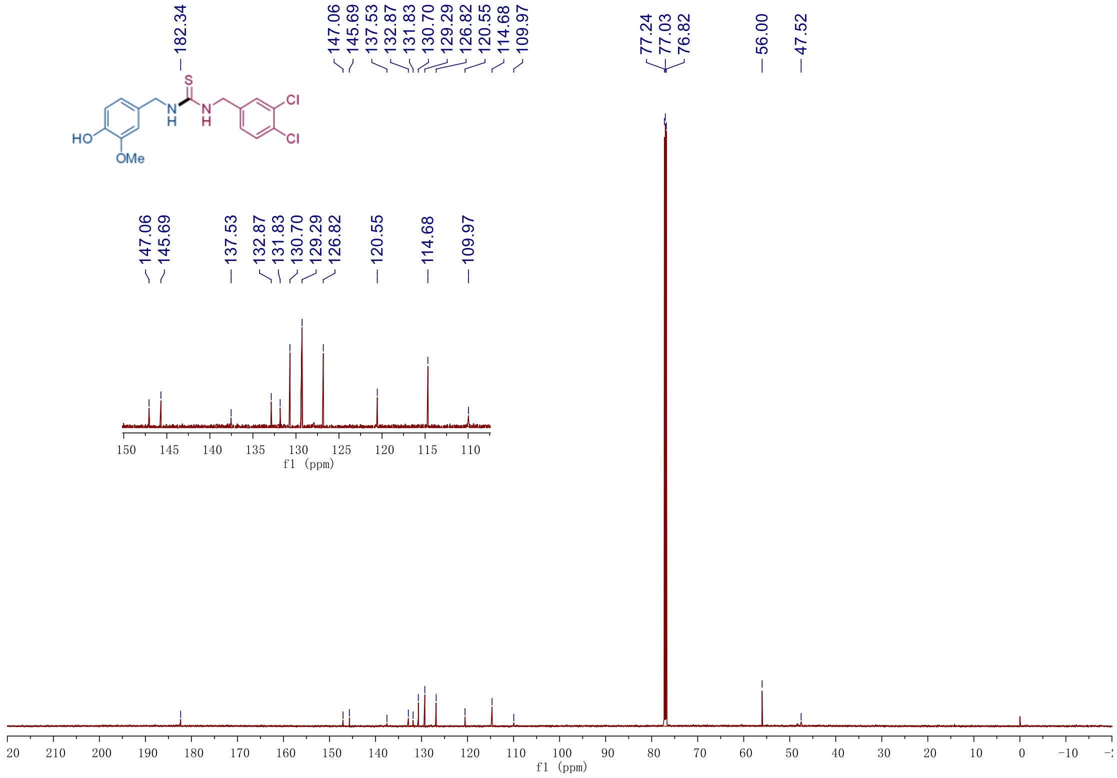

^{13}C NMR of compound **3i**

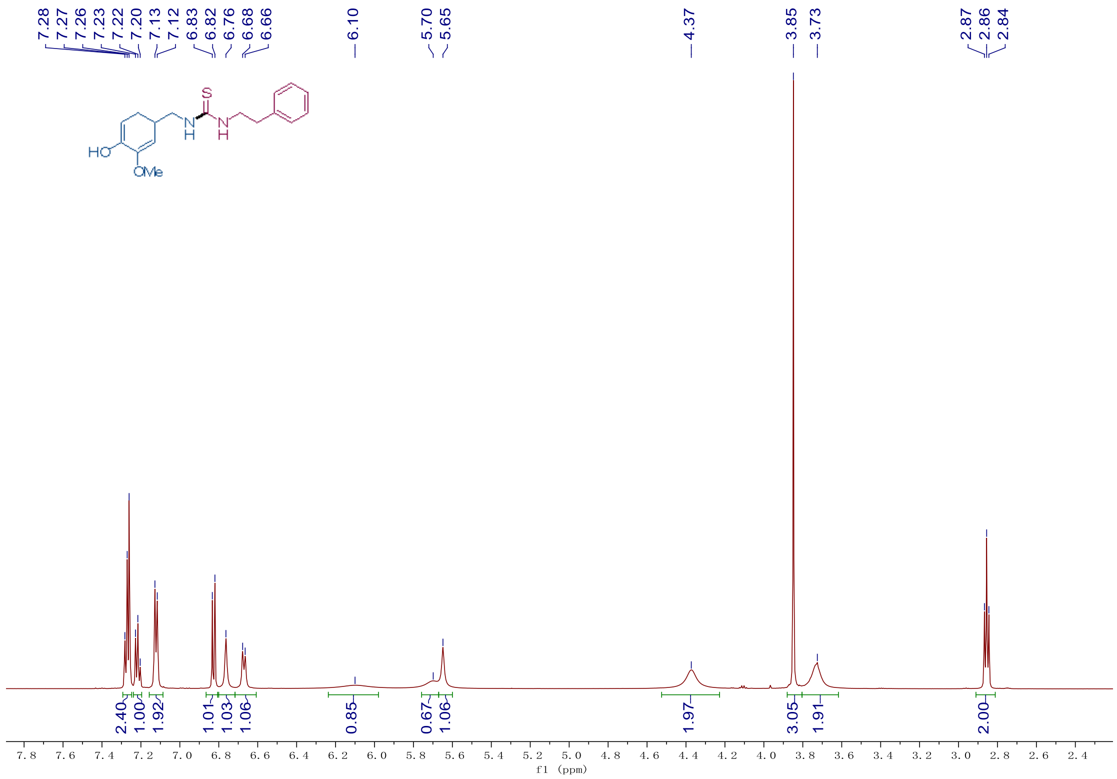

^{1}H NMR of compound **3j**

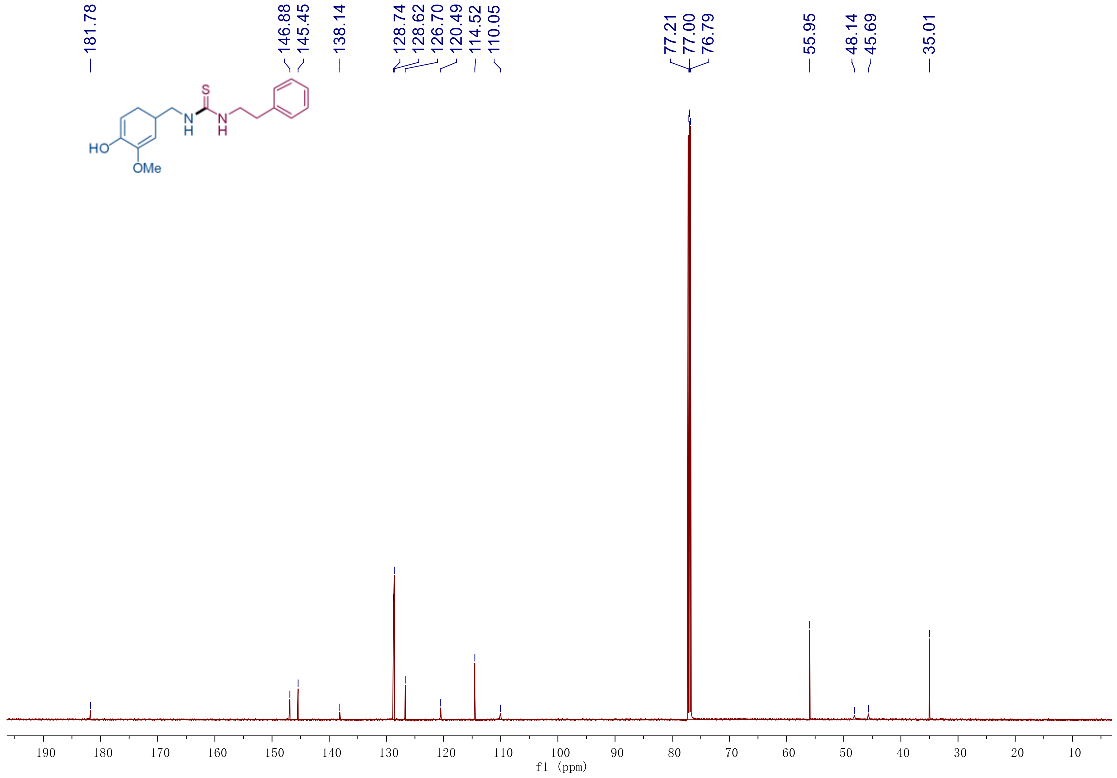

^{13}C NMR of compound **3j**

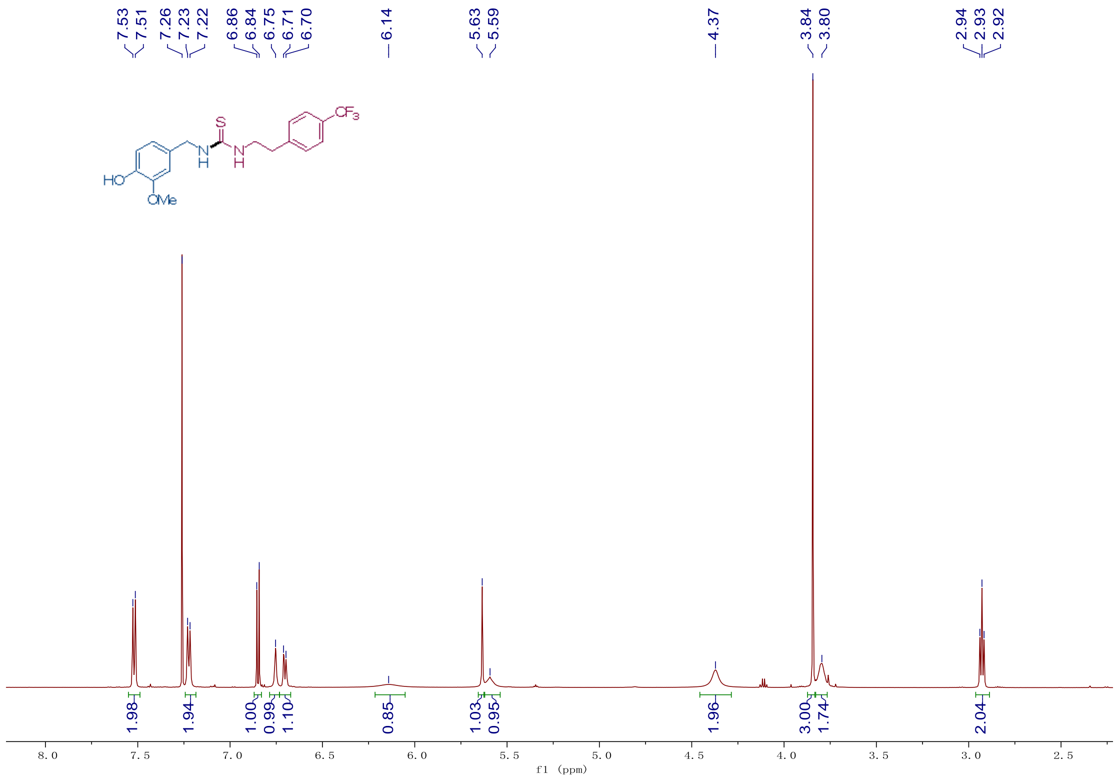

^{1}H NMR of compound **3k**

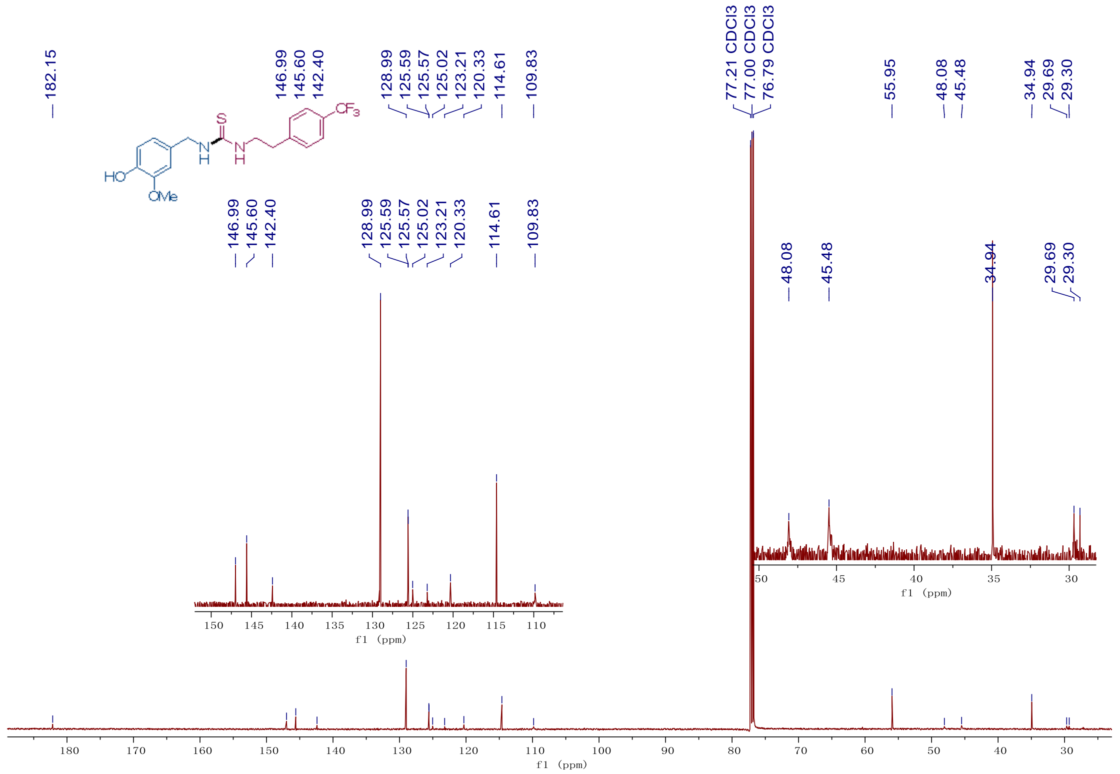

^{13}C NMR of compound **3k**

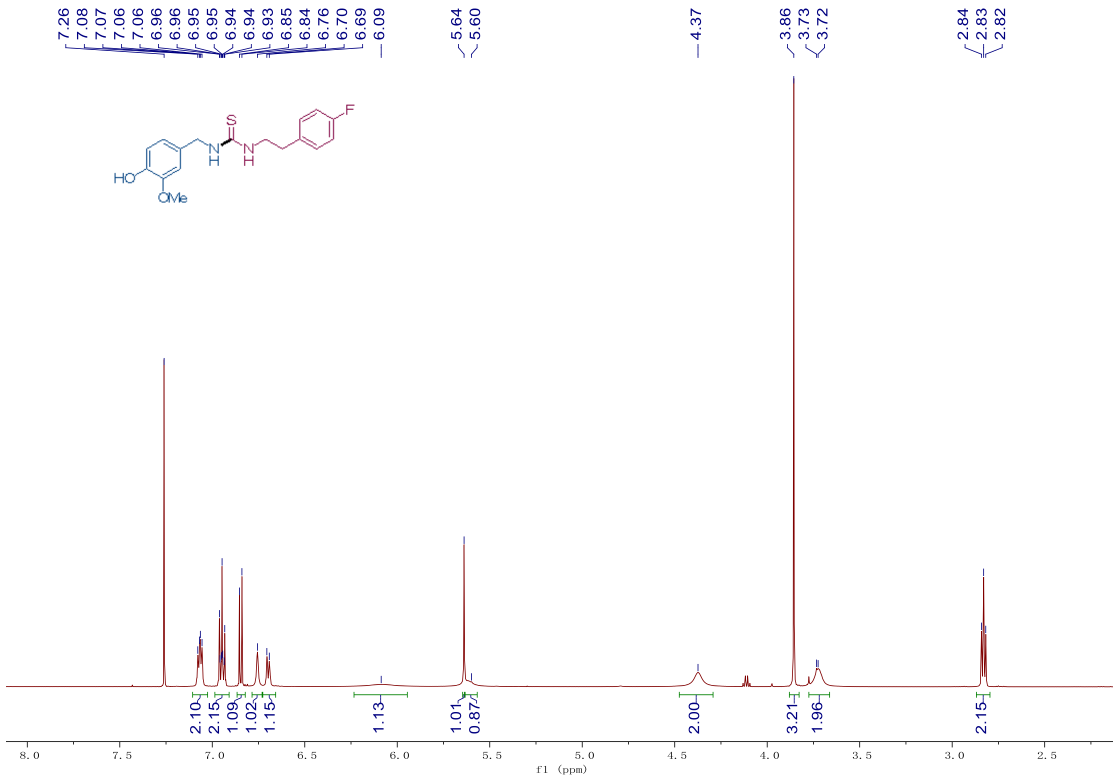

^{1}H NMR of compound **3l**

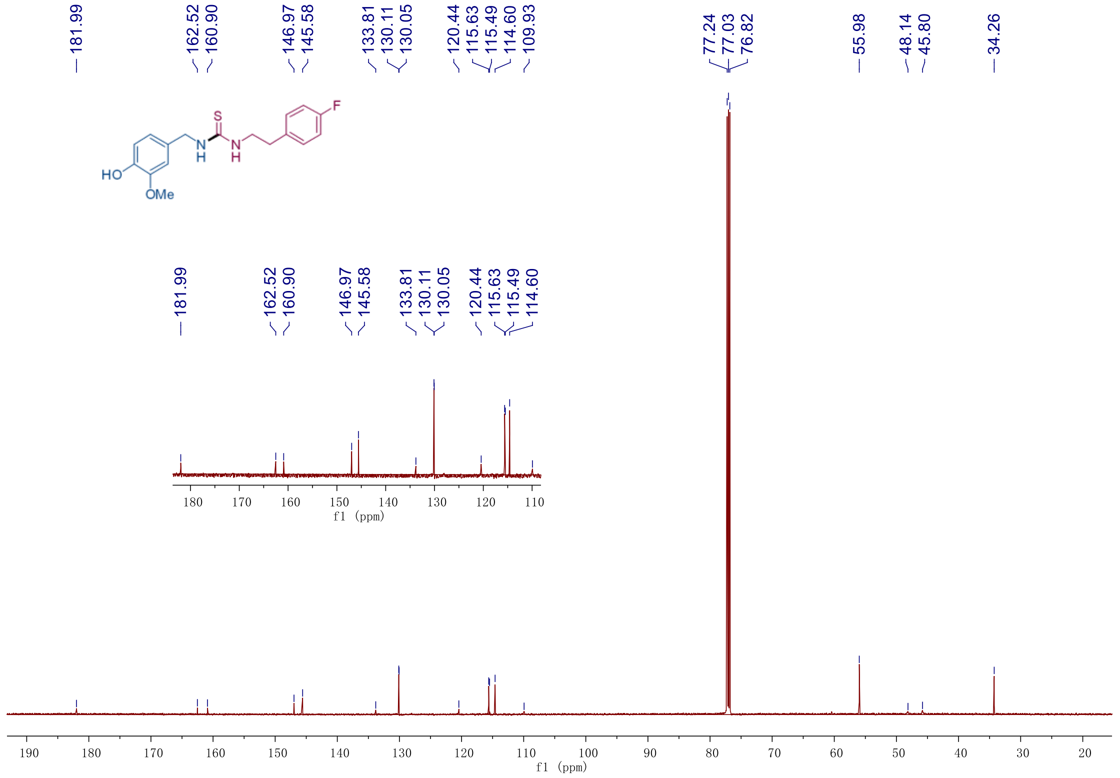

^{13}C NMR of compound **3l**

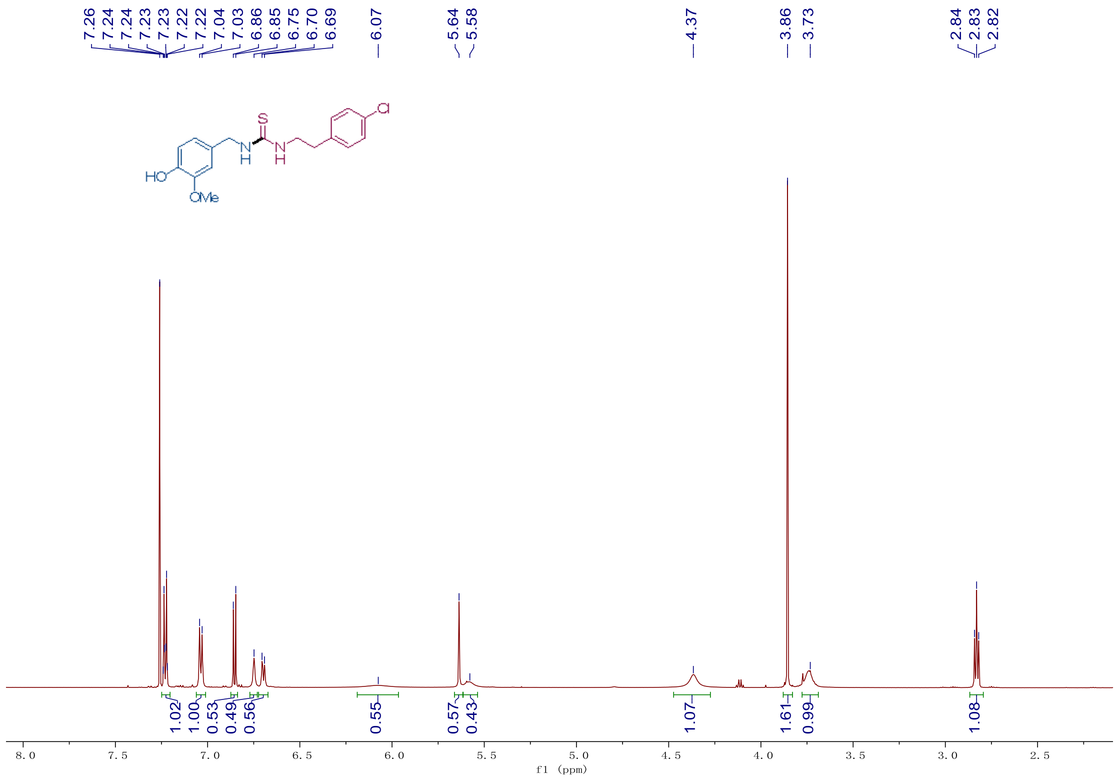

^{1}H NMR of compound **3m**

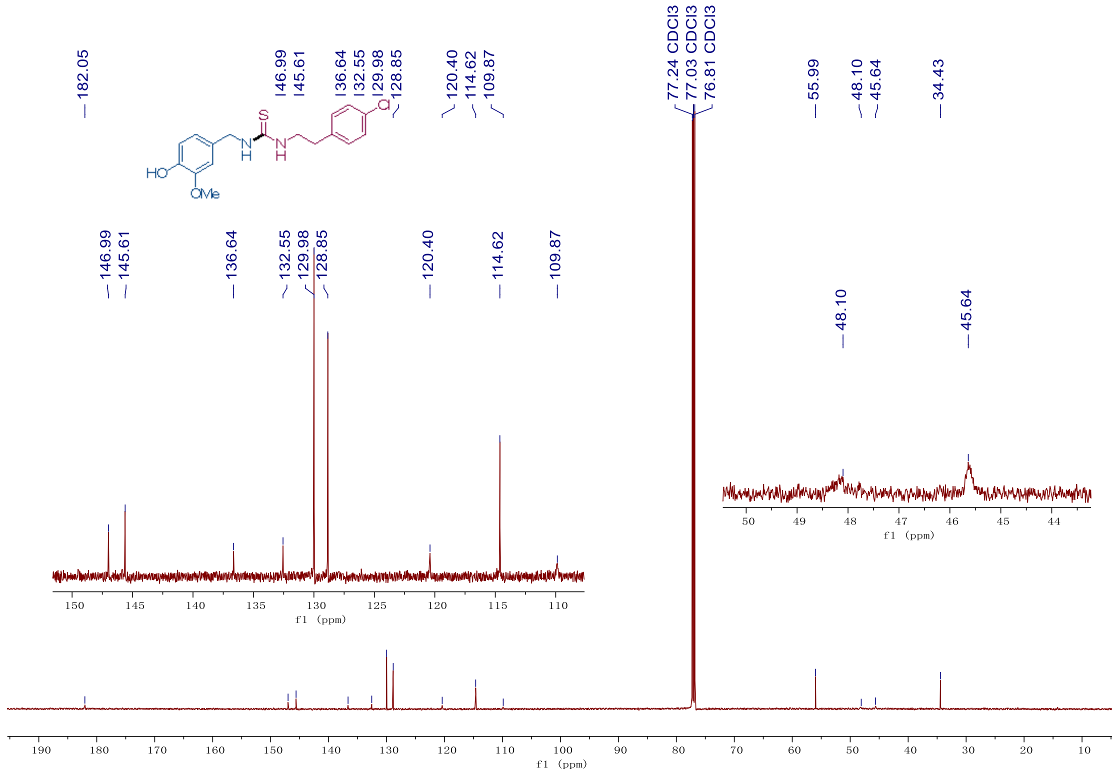

^{13}C NMR of compound **3m**

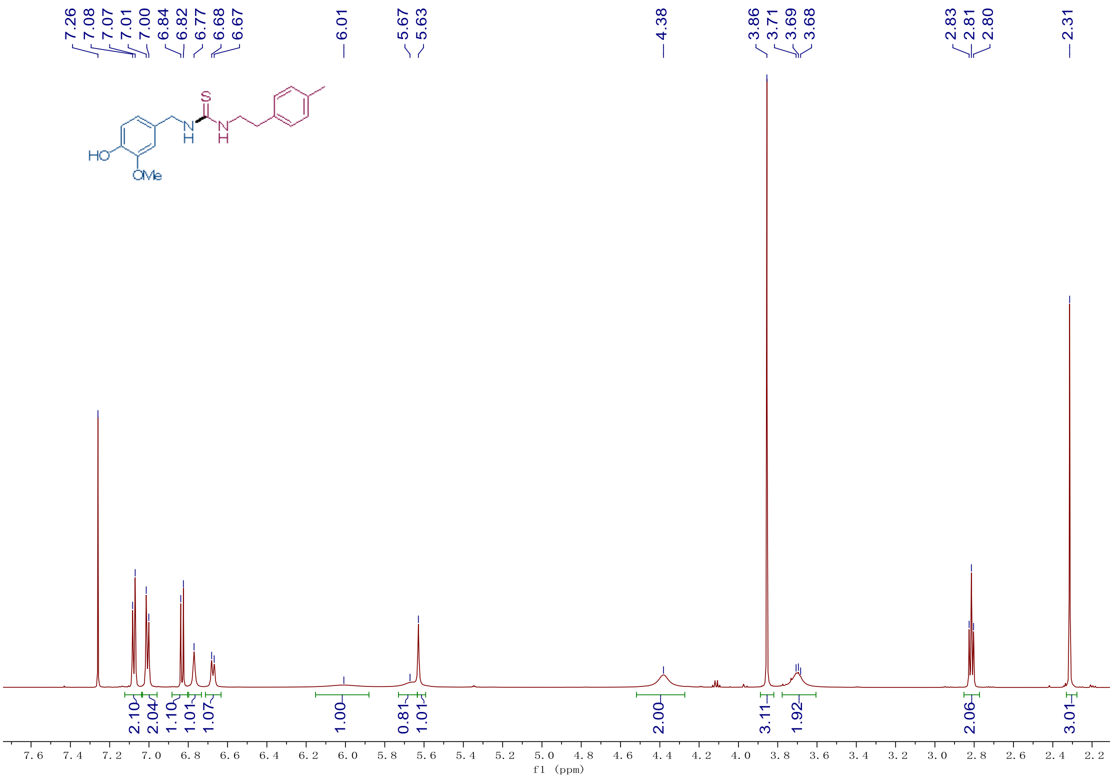

^{1}H NMR of compound **3n**

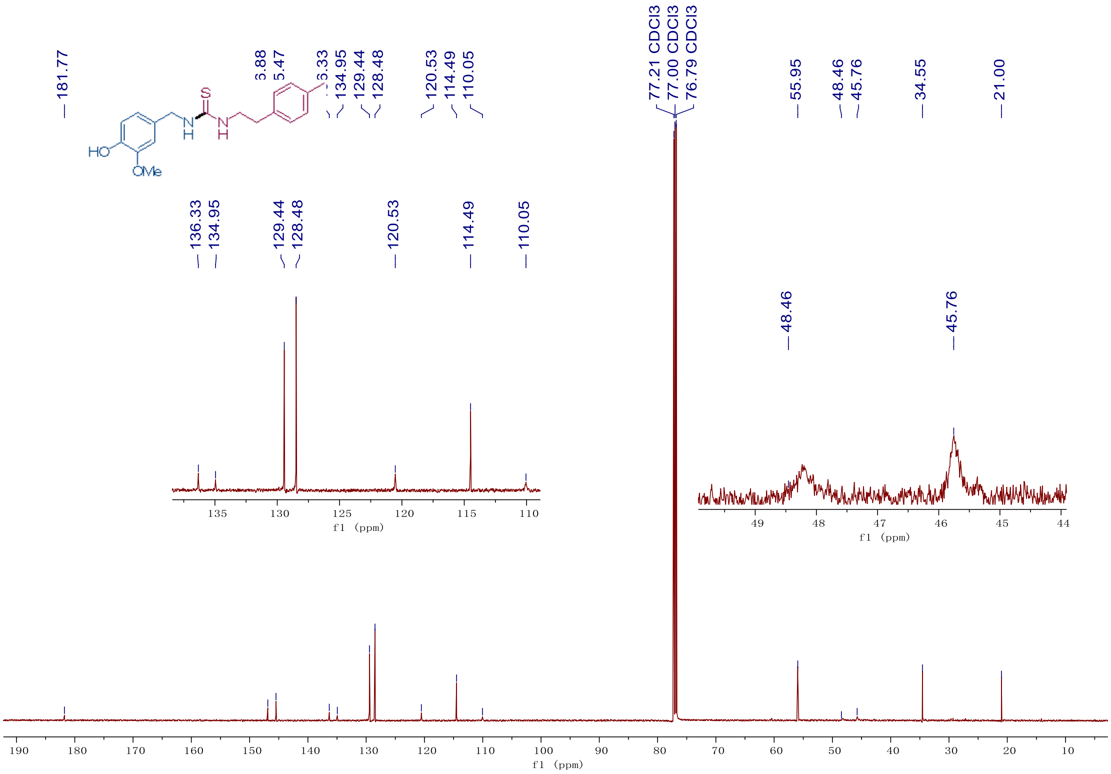

^{13}C NMR of compound **3n**

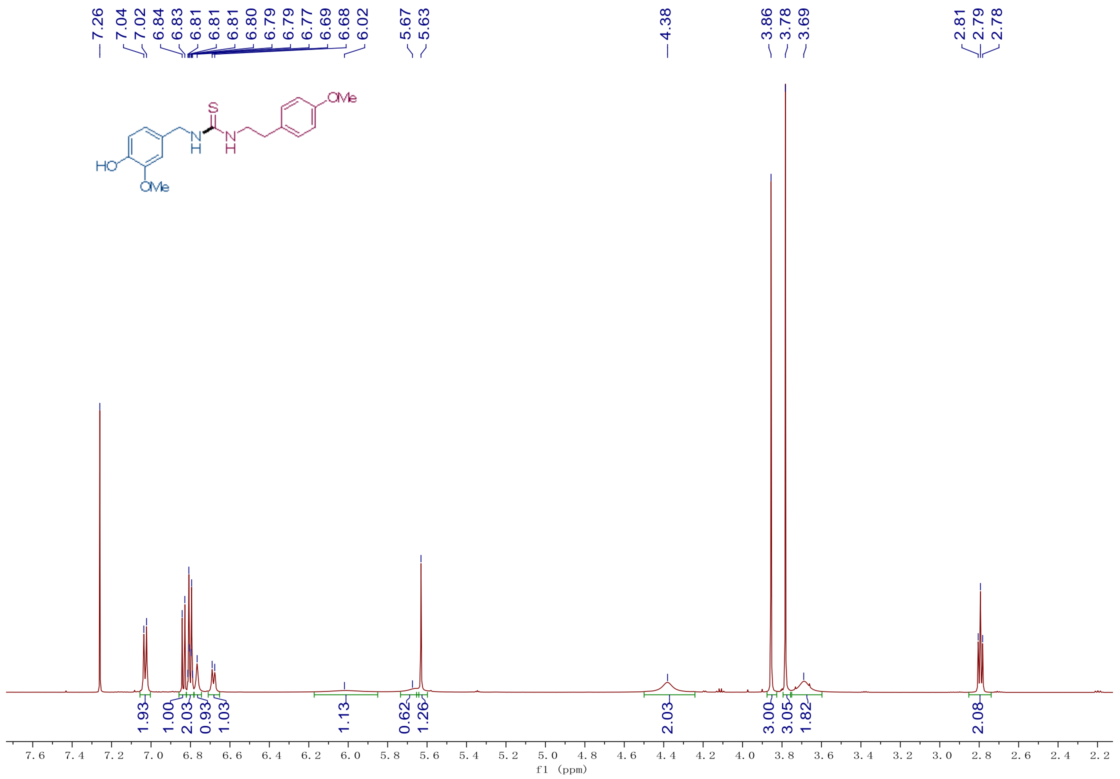

^{1}H NMR of compound **3o**

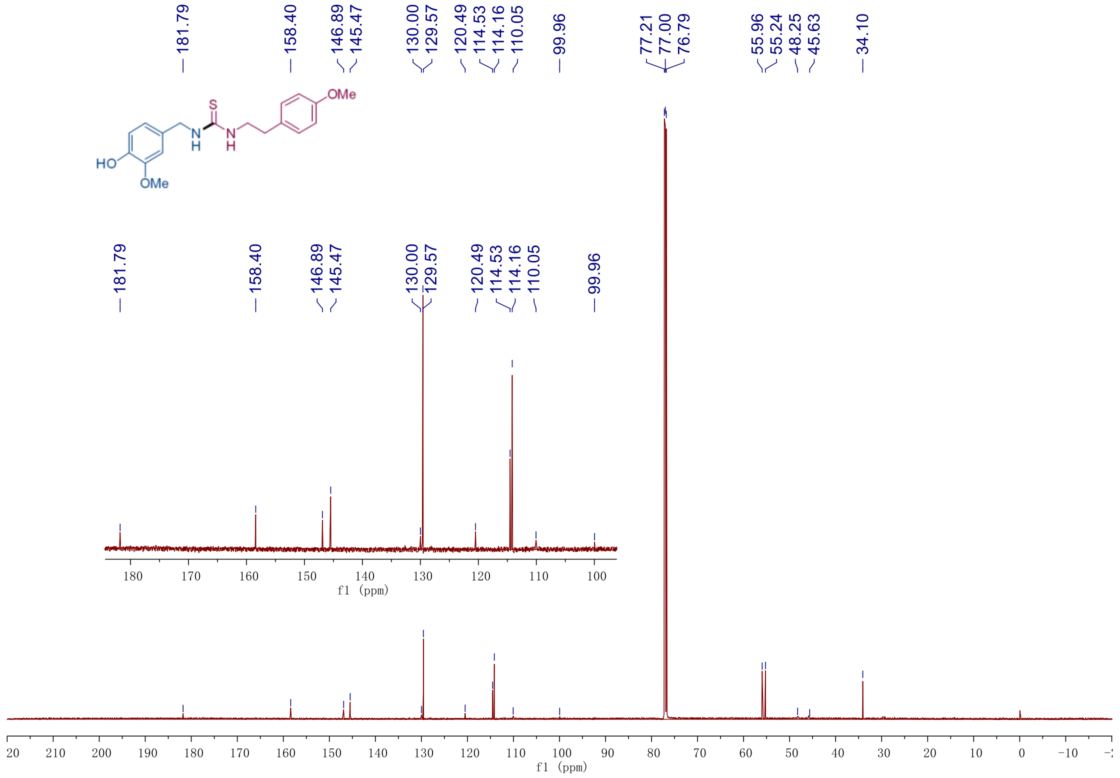

^{13}C NMR of compound **3o**

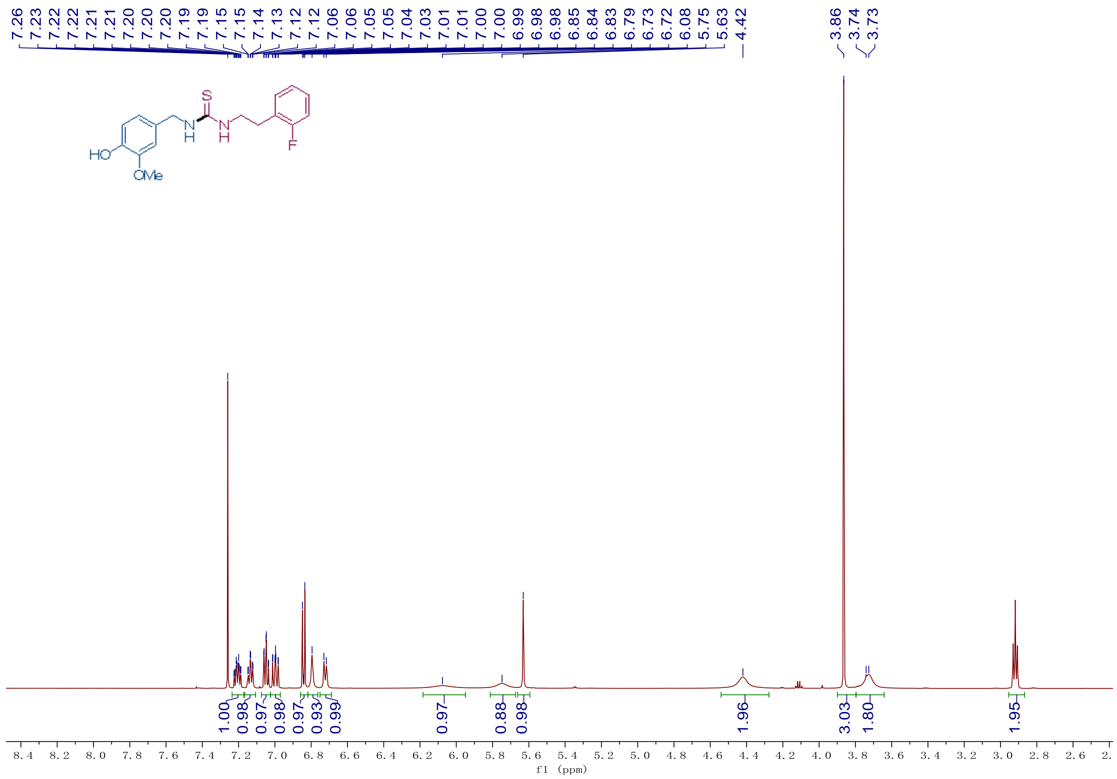

^{1}H NMR of compound **3p**

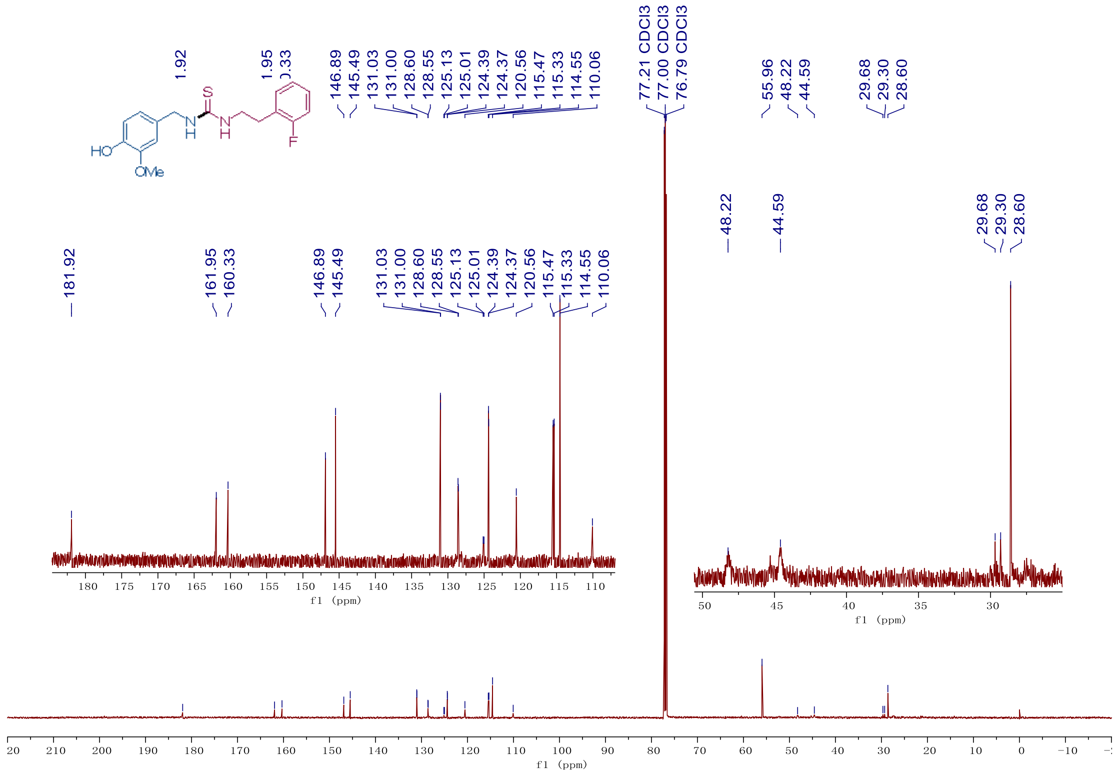

^{13}C NMR of compound **3p**

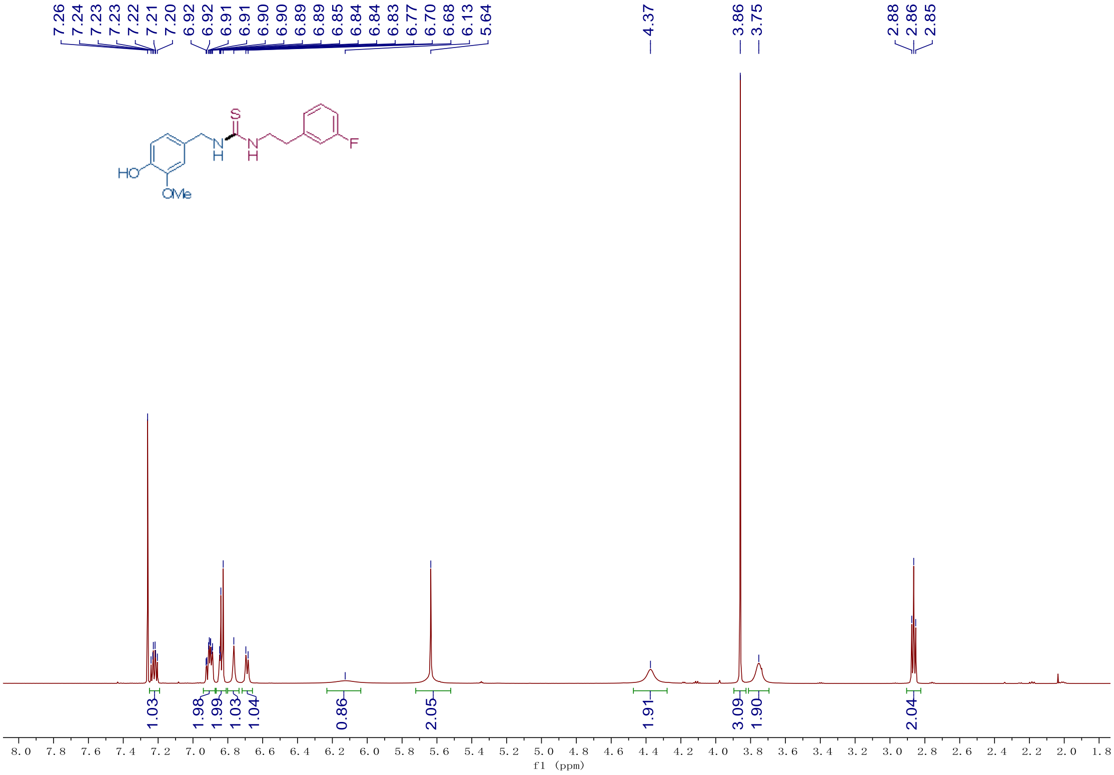

^{1}H NMR of compound **3q**

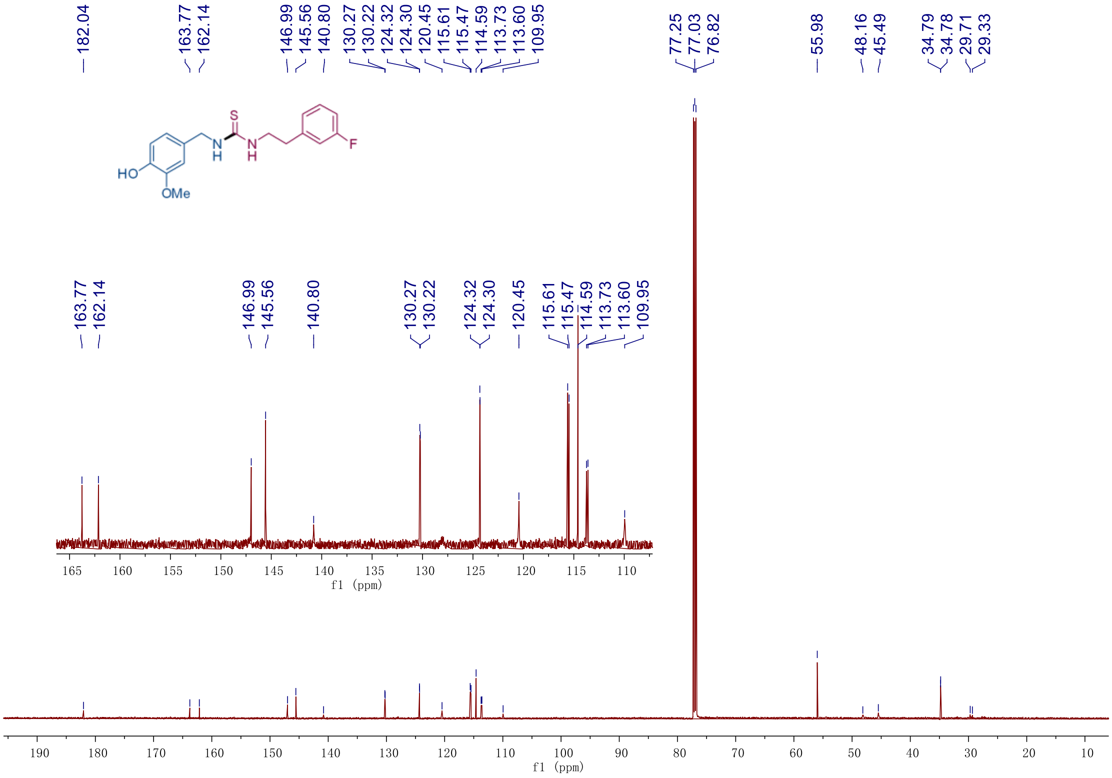

^{13}C NMR of compound **3q**

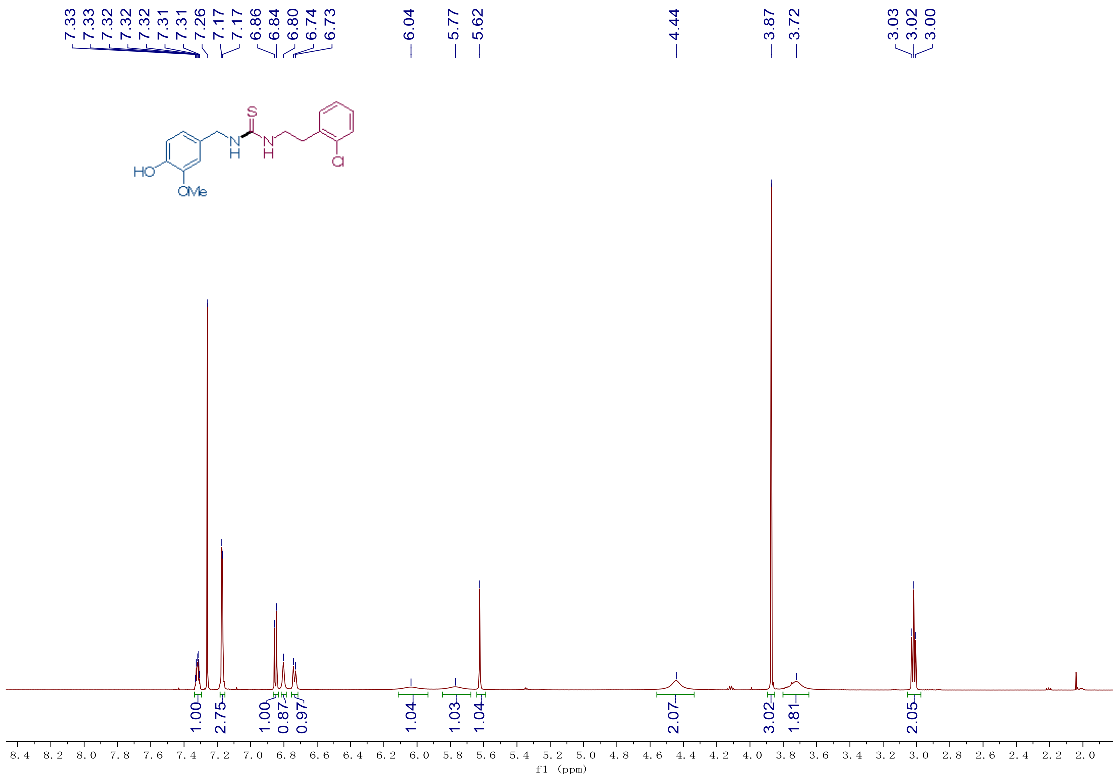

^{1}H NMR of compound **3r**

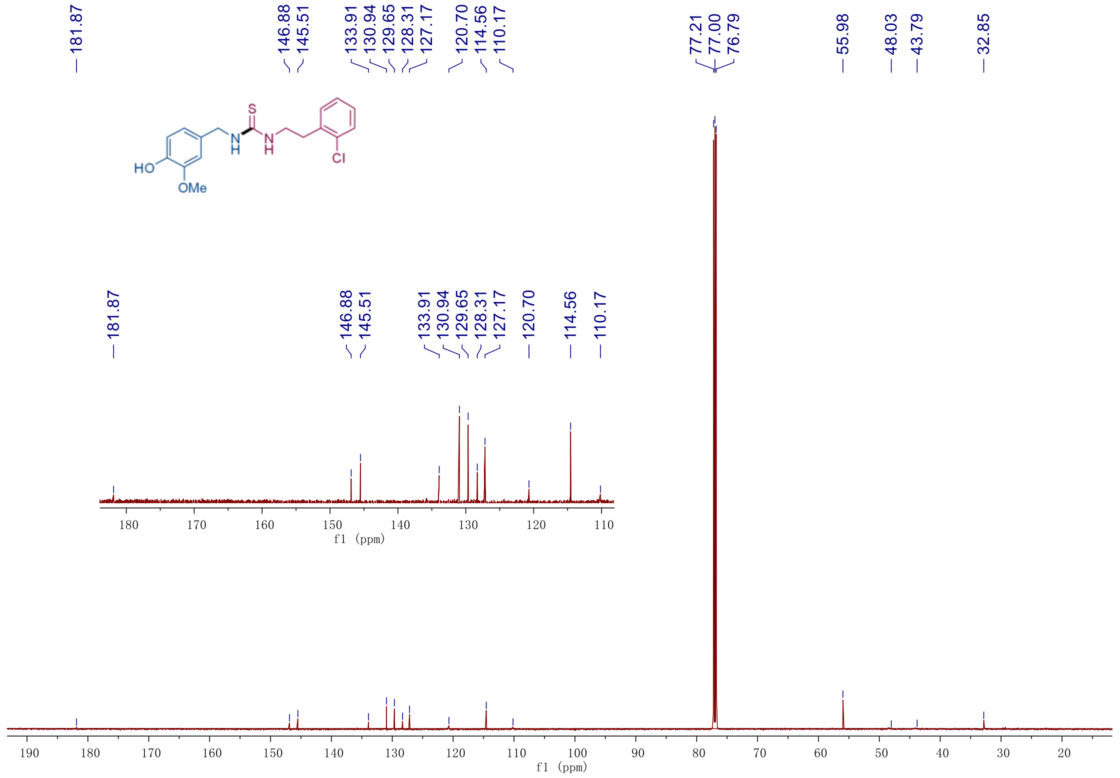

^{13}C NMR of compound **3r**

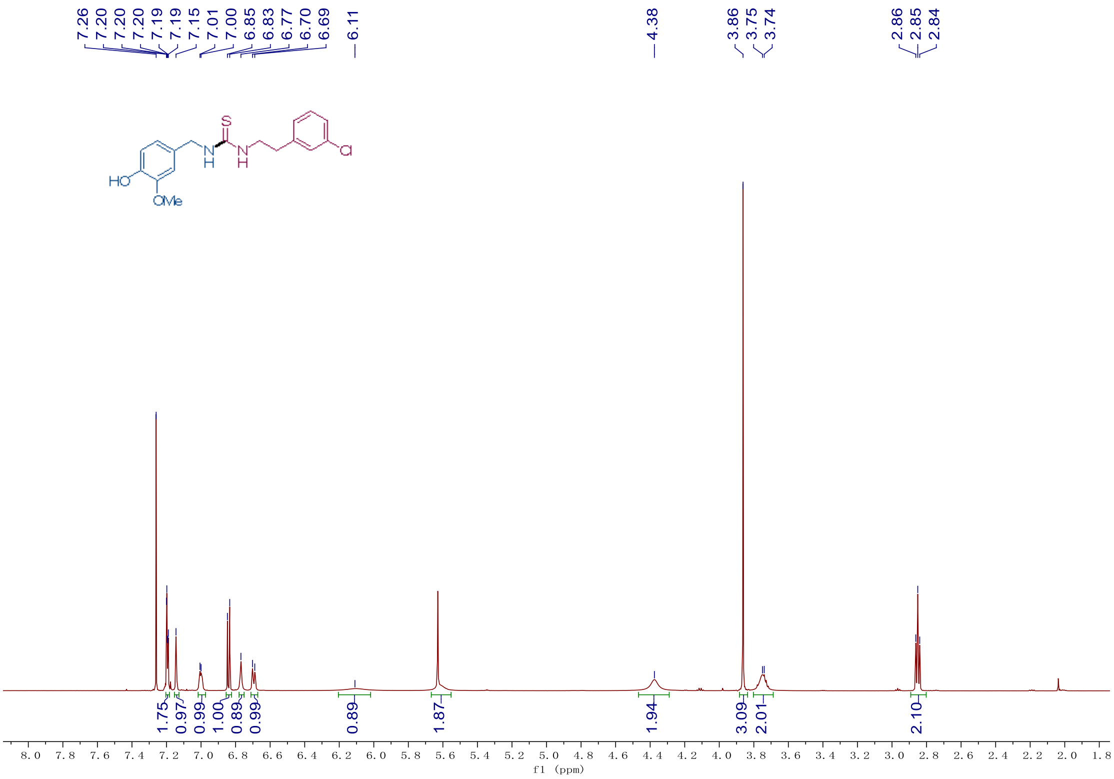

^{1}H NMR of compound **3s**

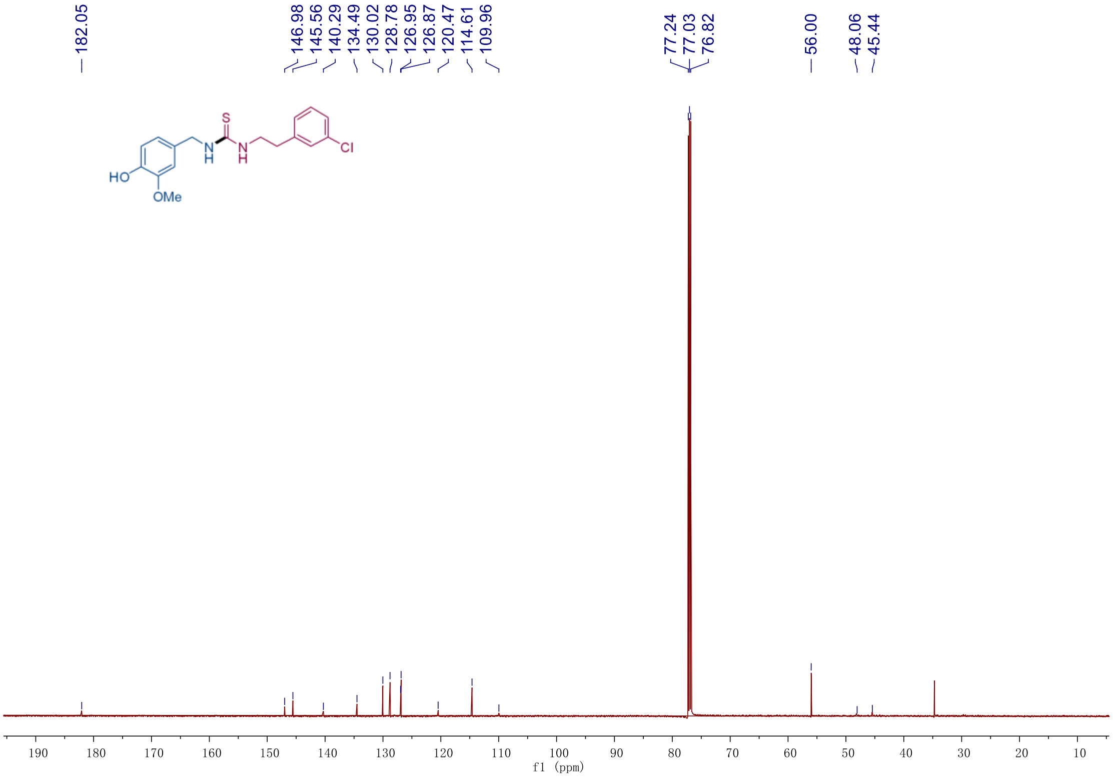

^{13}C NMR of compound **3s**

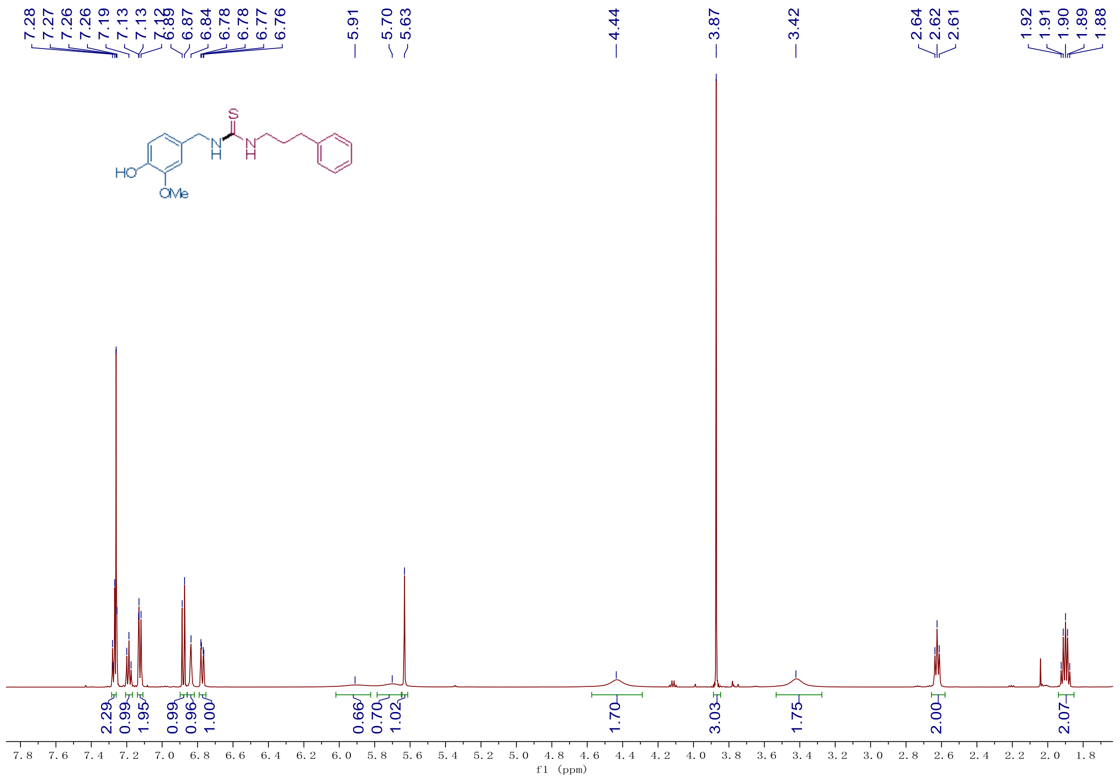

^{1}H NMR of compound **3t**

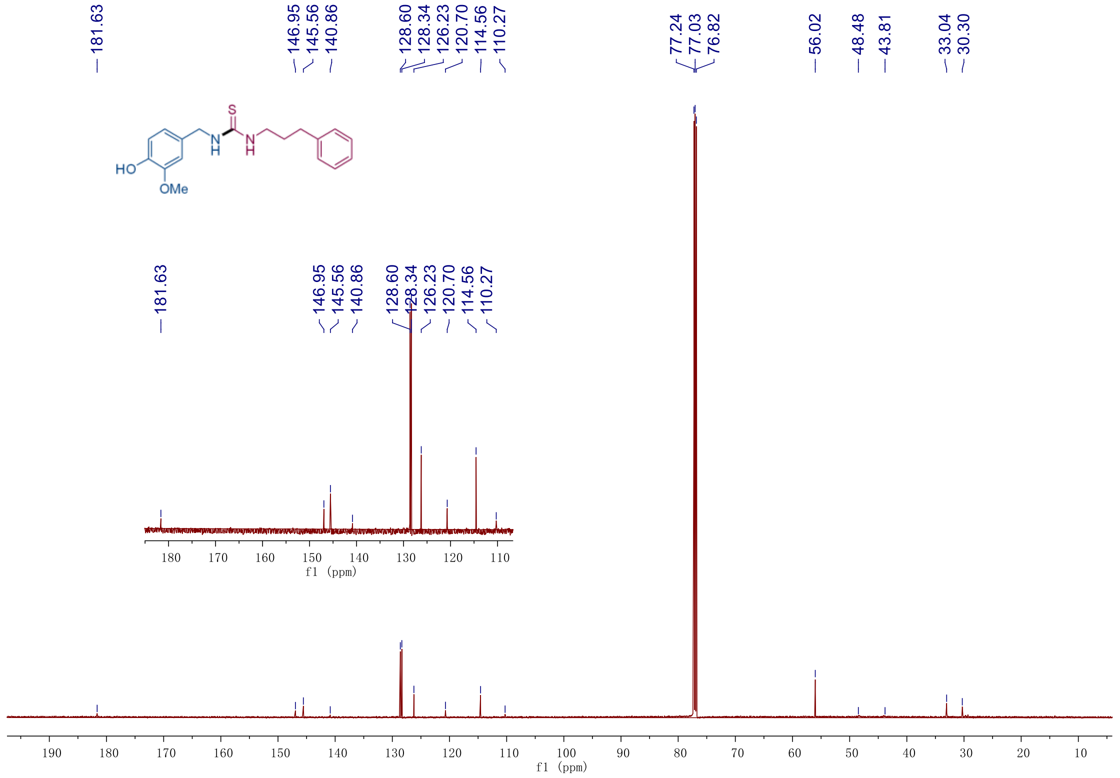

^{13}C NMR of compound **3t**

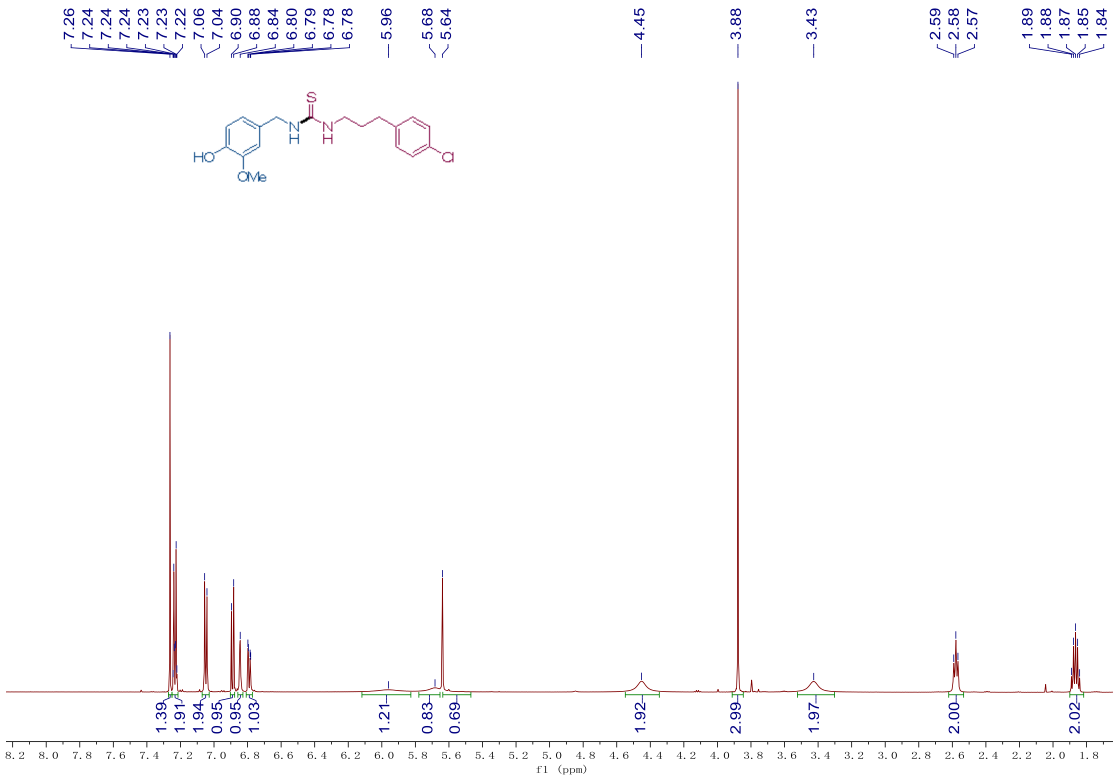

^{1}H NMR of compound **3u**

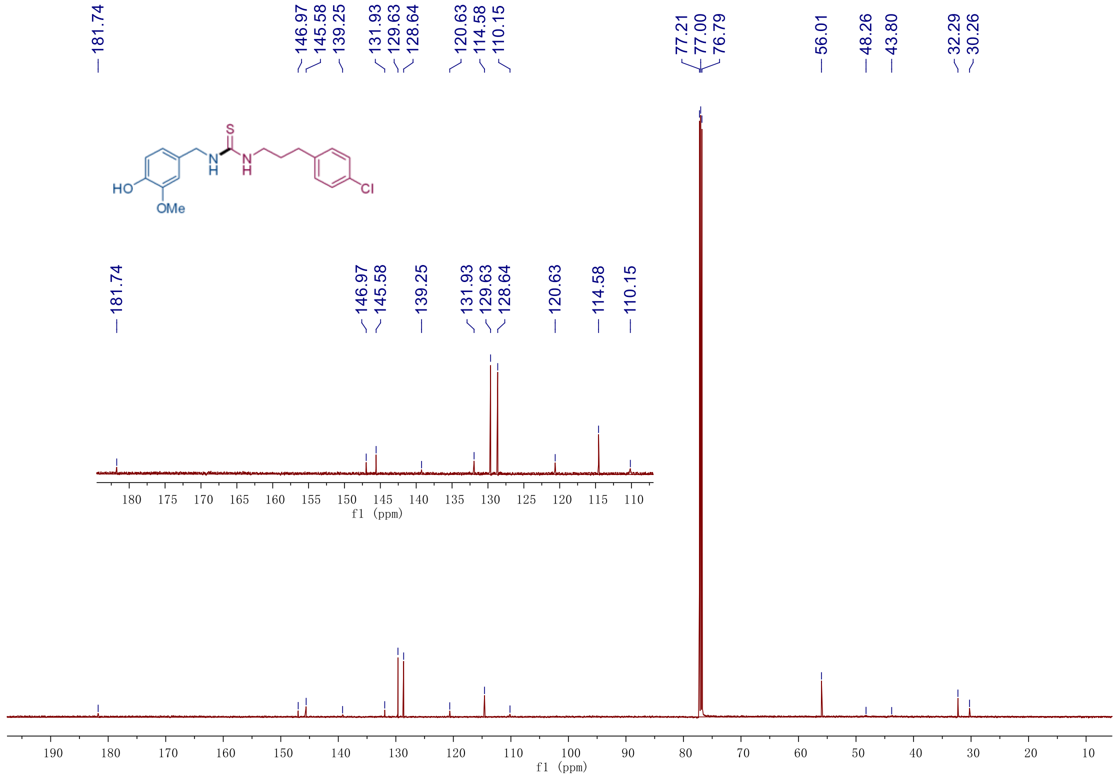

^{13}C NMR of compound **3u**

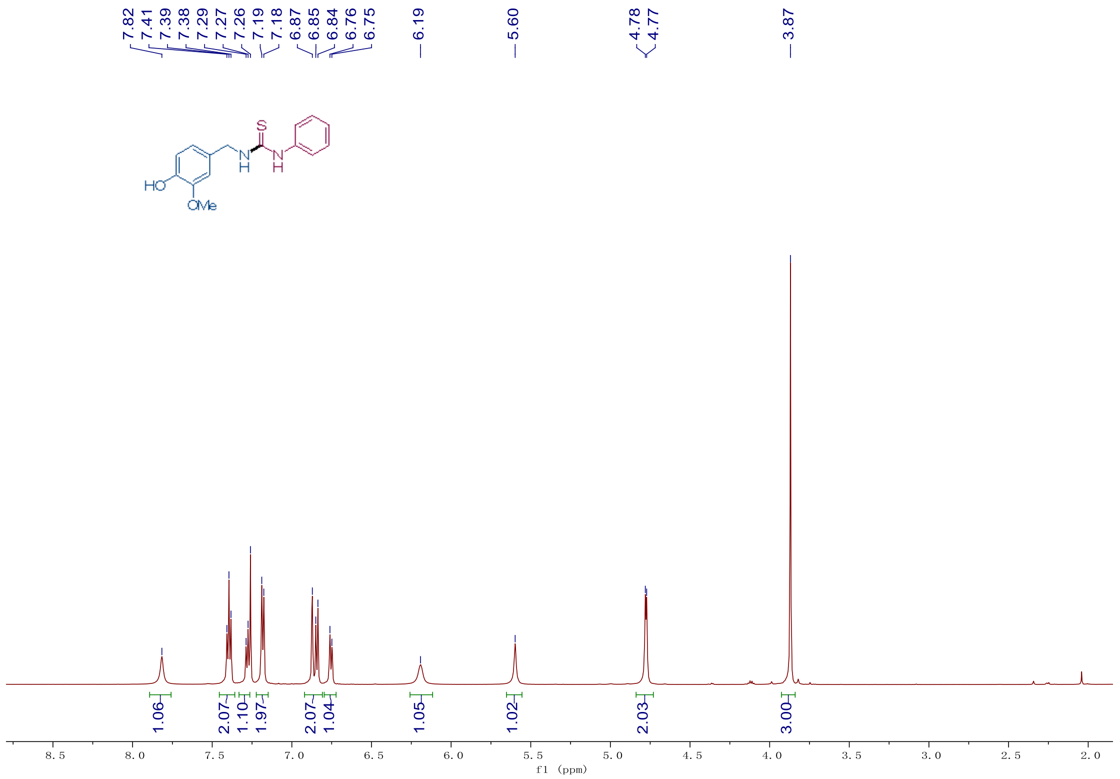

^{1}H NMR of compound **3v**

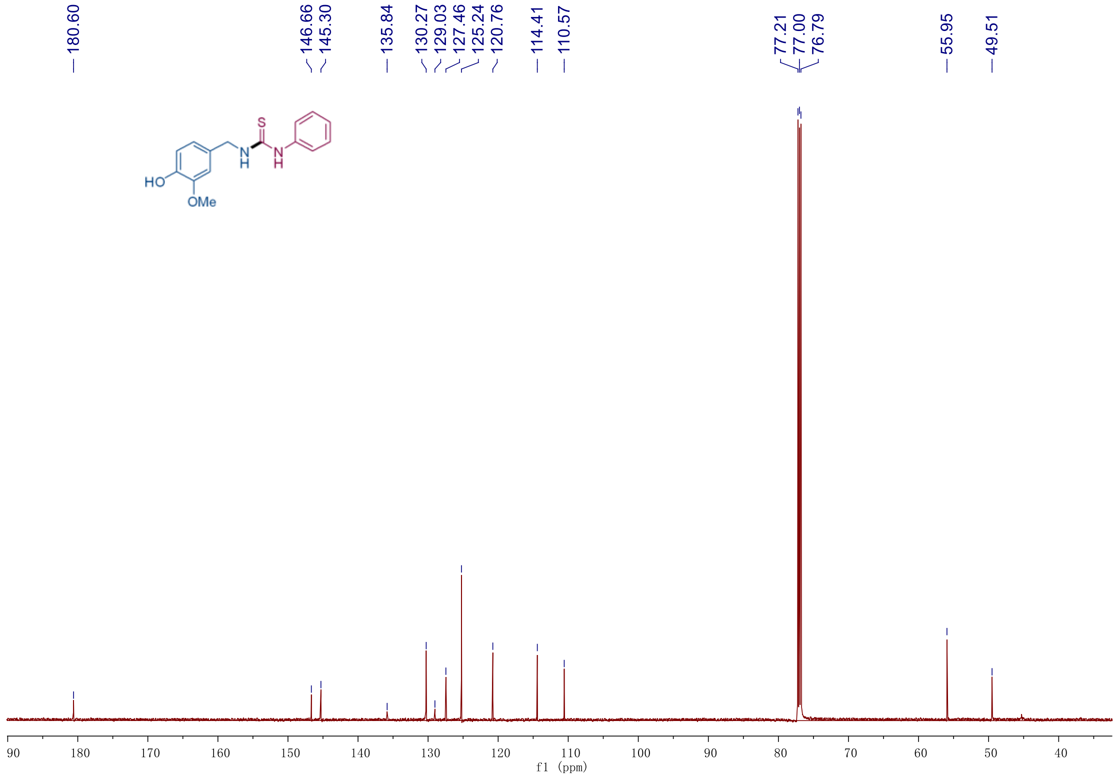

^{13}C NMR of compound **3v**

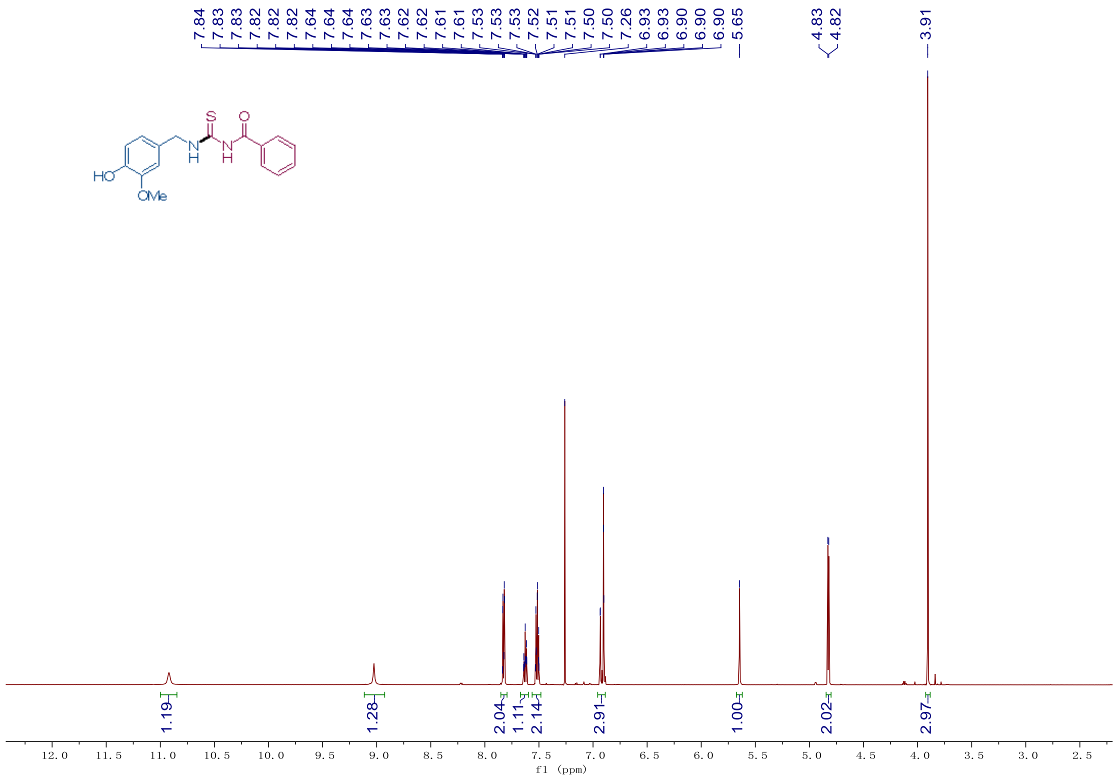

^{1}H NMR of compound **3w**

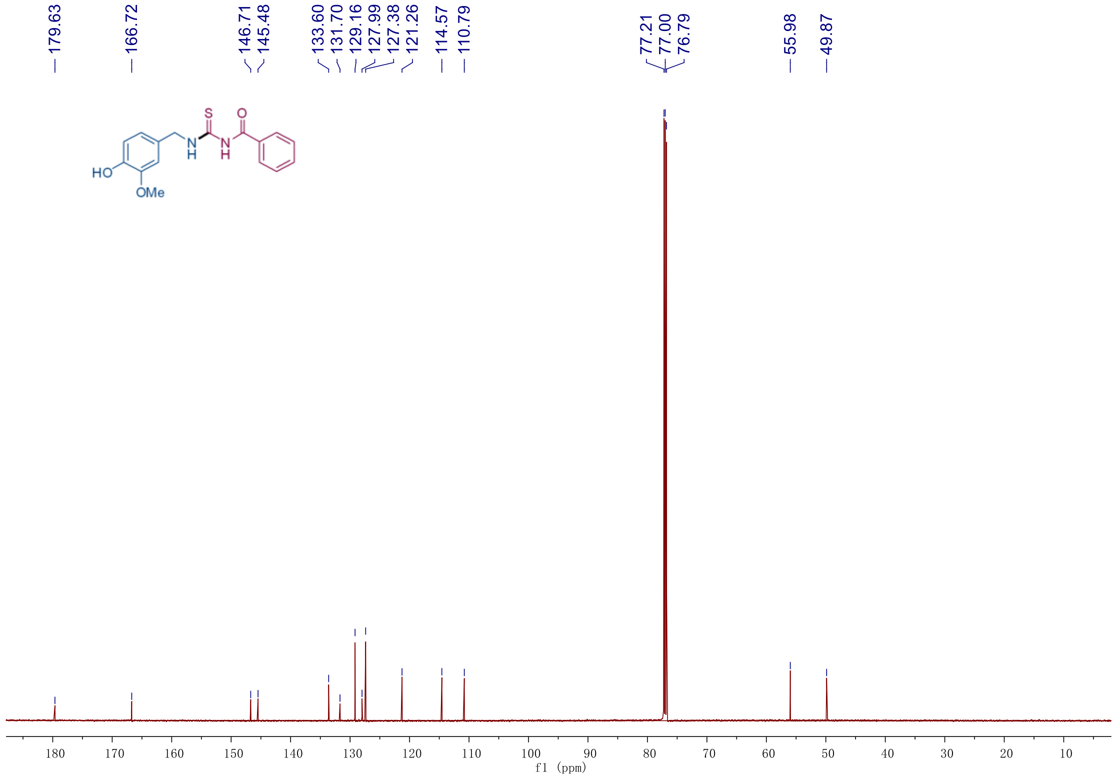

^{13}C NMR of compound **3w**

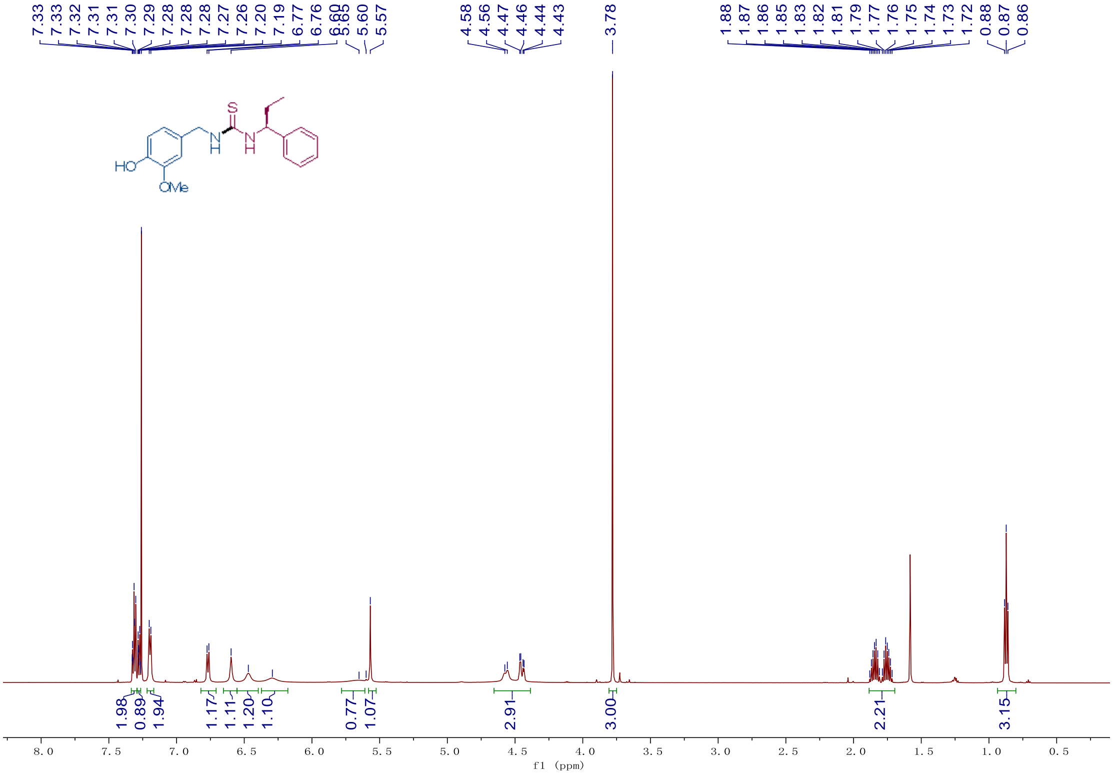

^{1}H NMR of compound **3x**

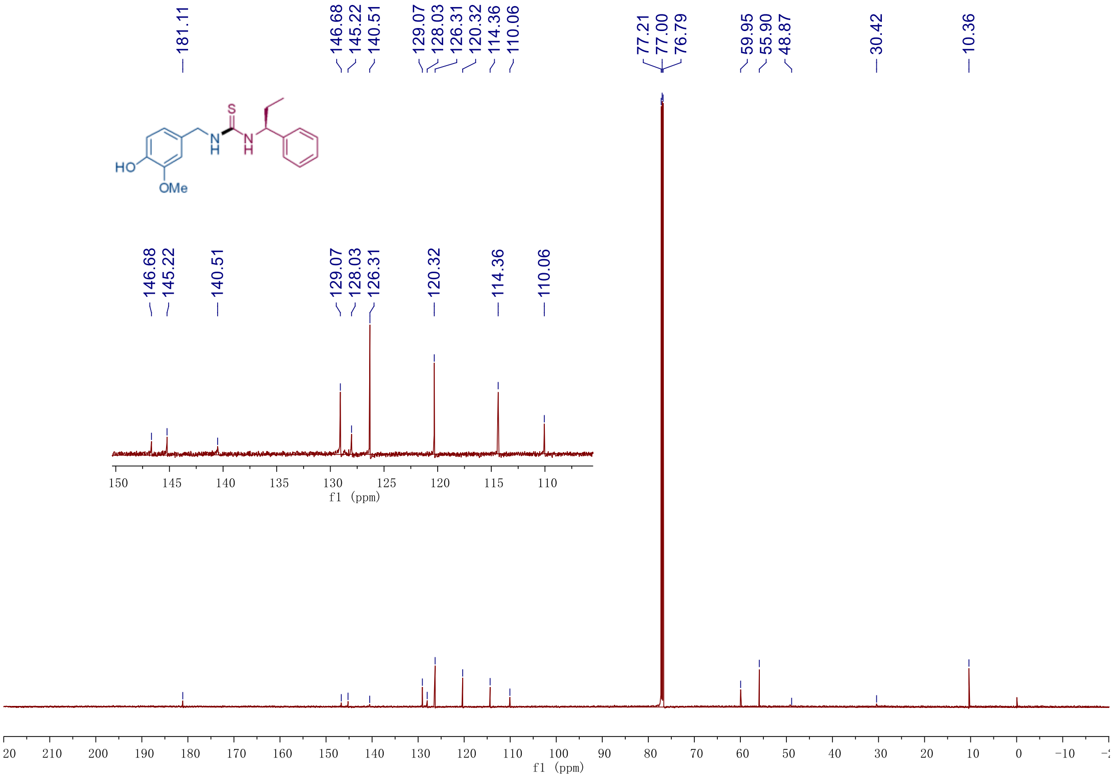

^{13}C NMR of compound **3x**

3. References

1 C. S. J. Walpole, R. Wrigglesworth, S. Bevan, E. A. Campbell, A. Dray, I. James, G. A. Hughes, K. J. Masdin, M. N. Perkins, J. Winter. *J. Med. Chem*., 1993, **36**, 2381.

2 R. Wrigglesworth, C. S. J. Walpole, S. Bevan, E. A. Campbell, A. Dray, G. A. Hughes, I. James, K. J. Masdin, J.

Winter. *J. Med. Chem.*, 1996, **39**, 4942.
